# Pangenome-Guided *In Silico* Design and Structural Evaluation of a Multi-Epitope Vaccine Candidate Against *Streptococcus suis*

**DOI:** 10.3390/ph19091448

**Published:** 2026-09-12

**Authors:** Nada Saleh Alhaggass, Waad A. Aljohani, Reem Alromaihi, Sarah Nasser Alnuwaysir, Razan Abdalrahman Almohimid, Ahmad Almatroudi, Khaled S. Allemailem

**Affiliations:** 1 Department of Biology, College of Science, Qassim University, Buraydah 51452, Saudi Arabia; 2 Department of Basic Health Sciences, College of Applied Medical Sciences, Qassim University, Buraydah 51452, Saudi Arabia; wa.aljohani@qu.edu.sa; 3 Department of Medical Laboratories, College of Applied Medical Sciences, Qassim University, Buraydah 51452, Saudi Arabia; r.alromaihi@qu.edu.sa (R.A.); 421201929@qu.edu.sa (S.N.A.); 421201931@qu.edu.sa (R.A.A.); aamtrody@qu.edu.sa (A.A.)

**Keywords:** *Streptococcus suis*, subtractive proteomics, immunoinformatics, molecular docking, molecular dynamic simulation, antimicrobial resistance, reverse vaccinology, multi-epitope vaccine

## Abstract

**Background/Objectives:** *Streptococcus suis* is an important zoonotic pathogen responsible for severe infections in animals and humans, and the emergence of diverse strains has reduced the effectiveness of conventional antimicrobial therapies. Since there is no broadly protective vaccine, there is a need for new vaccination strategies that focus on conserved antigens from a variety of strains. This study aimed to design and evaluate a multi-epitope vaccine candidate against diverse *S. suis* strains using an integrated pangenome-guided reverse vaccinology approach. **Methods:** To design a multi-epitope vaccine (MEV) candidate against diverse *S. suis*, an integrated computational framework was employed, incorporating pangenome analysis, subtractive proteomics, reverse vaccinology, immunoinformatics, structural modeling, molecular docking, molecular dynamics simulation, immune simulation, and *in silico* cloning. The conserved core proteins were systematically screened for essential, non-homologous, antigenic, non-allergenic and non-toxic vaccine candidates for epitope prediction. **Results:** A total of 7421 gene families, including 1169 conserved core genes, were identified through pangenome analysis of 24 complete *S. suis* genomes. Three computationally prioritized candidate proteins were identified through sequential subtractive proteomics: sucrose phosphorylase, peptidoglycan hydrolase PcsB and an RND transporter-associated adaptor protein, annotated in the source database as an RND efflux transporter periplasmic adaptor subunit. We selected eight cytotoxic T-lymphocyte (CTL) epitopes, five helper T-lymphocyte (HTL) epitopes, and three linear B-cell epitopes with favorable predicted immunological properties to develop a 397-amino acid multi-epitope vaccine construct that contains the *S. suis* 50S ribosomal protein L7/L12 adjuvant with rationally designed peptide linkers. The vaccine construct exhibited favorable physicochemical properties, predicted structural stability, and high antigenicity scores. The predicted combined HLA population coverage of the selected CTL and HTL epitopes was 90.77% across the populations included in the analysis. Immune simulation predicted patterns consistent with humoral and cellular immune activation, including sustained IgG production, elevated IFN-γ and IL-2 secretion, efficient antigen clearance, and generation of immunological memory, whereas molecular docking and molecular dynamics simulations characterized the predicted interaction and conformational behavior of the MEV–TLR1/TLR2 complex. Codon optimization (CAI = 0.996) and *in silico* cloning into the pET-30a(+) expression vector supported the potential feasibility of recombinant expression in *Escherichia coli*. **Conclusions:** In this study, a rationally designed multi-epitope vaccine candidate against diverse *S. suis* strains was developed using an integrated pangenome-guided reverse vaccinology approach. Based on these computational analyses, the proposed vaccine candidate showed favorable predicted immunogenicity, predicted structural quality, predicted HLA population coverage, and expression feasibility, providing a foundation for future experimental validation and development of a vaccine against diverse *S. suis*.

## 1. Introduction

Antimicrobial resistance (AMR) has shifted from a looming concern to a present and worsening reality for global health. A comprehensive systematic analysis of the global burden of bacterial AMR estimated that resistant infections were associated with close to five million deaths in 2019 alone and projected that, without effective intervention, annual AMR-related mortality could rise by nearly 70% by 2050 [1,2]. Diverse bacterial pathogens are now considered among the most pressing threats to modern medicine, steadily narrowing the antibiotic arsenal available to clinicians and complicating the management of infections once considered routine [3,4]. Vaccination is increasingly recognized as a direct lever against this trend rather than simply a complement to antimicrobial therapy: by preventing infection outright, vaccines reduce the number of episodes for which antibiotics are prescribed, curb the onward transmission of resistant clones within both human and animal populations, and lower the selective pressure that drives the emergence of new resistance determinants, effects that have been explicitly incorporated into “reverse vaccine development” strategies aimed specifically at AMR pathogens [5,6].

*S. suis* illustrates this convergence of zoonotic risk and antimicrobial resistance with particular clarity. For decades regarded as a pathogen of primarily veterinary and agricultural significance, *S. suis* is now firmly established as a genuine human pathogen, most often causing meningitis but also septicemia, endocarditis, arthritis, and streptococcal toxic-shock-like syndrome, typically following occupational or dietary exposure to its animal reservoir [7,8]. The human toll is not trivial: pooled estimates from systematic reviews place the case-fatality rate of *S. suis* meningitis at roughly 3–13%, yet hearing loss, the pathogen’s signature sequela, affects close to half of survivors, a burden of permanent disability rarely seen with other causes of bacterial meningitis [9]. In Thailand alone, a health-economic model estimated a substantial loss of disability-adjusted life years once productivity losses among working-age men were accounted for [10], and the disease imposes a parallel economic burden on the animal agriculture sector through animal mortality, reduced growth performance, and product condemnation. Systematic surveillance has further shown that human *S. suis* infections are substantially underreported even in regions with well-developed diagnostic infrastructure, with more than two hundred cases identified across seven European reference laboratories in a single retrospective survey [7]. The geographic reach of the pathogen continues to expand beyond its traditional strongholds in East and Southeast Asia: a recent review of its evolutionary and epidemiological trajectory documented extensive serotype and sequence-type diversity alongside a marked rise in multidrug resistance among clinical isolates [8], while newly compiled case series have confirmed an emerging, still under-recognized burden of human infection across South America [11]. Clinical management is further complicated by a demonstrated decline in penicillin susceptibility among isolates from recent hospital-based cohorts [12] and, in many, though not all strains, by the capacity to form biofilms. Biofilm-forming ability in *S. suis* is in fact highly heterogeneous, varying considerably across serotypes and even among strains of the same serotype, but where present it is associated with enhanced persistence and antibiotic tolerance, as it is in bacteria more generally [13,14].

This combination of clinical severity, expanding geographic reach, and eroding antibiotic efficacy means that conventional, narrowly targeted approaches to *S. suis* control are increasingly unlikely to keep pace with the pathogen on their own, and it is this shortfall that has pushed antigen discovery toward more systematic, genome-scale strategies. Despite the clinical importance the field has long recognized, no broadly protective vaccine against *S. suis* is currently licensed for use in humans or in the animal populations that constitute its natural reservoir, and the reasons are reasonably well understood. The pathogen’s extensive antigenic and serotype diversity, comprising more than 29 recognized serotypes and well over a thousand sequence types, means that vaccines built around a small number of strain-specific antigens, whether whole-cell bacterins or single recombinant proteins, tend to protect narrowly against the serotypes from which they were derived rather than across the population as a whole; immune responses restricted to a single arm of immunity, typically antibody alone, have also generally proved insufficient against a pathogen capable of intracellular survival and immune evasion [15,16]. In the absence of a licensed product, autogenous bacterins prepared from isolate-specific strains have become the default control measure in parts of Europe, but these preparations are rarely subjected to rigorous safety or efficacy testing, and the protection they confer is generally restricted to the strains from which they were derived [17]. Immunoinformatics-guided multi-epitope vaccine (MEV) design has begun to address this gap: recent studies have assembled epitopes from conserved, virulence-associated *S. suis* surface proteins into single chimeric constructs, reporting favorable predicted antigenicity and structural stability [15], and, in at least two instances, meaningful cross-serotype protection when tested in murine models [18,19]. Rather than claiming methodological novelty for pangenome-guided reverse vaccinology or multi-epitope vaccine design themselves, the present study applies an integrated workflow in which the conserved core proteome of 24 complete *S. suis* genomes serves as the starting point for sequential subtractive proteomics, epitope prioritization, structural assessment, receptor docking, molecular dynamics, immune simulation, and *in silico* expression analysis. The principal contribution is therefore the specific combination and application of these computational filters to prioritize vaccine candidates from the selected genome set, including an RND-associated protein that requires further biological validation.

A multi-epitope design was chosen deliberately rather than as a matter of convenience. Combining MHC class I-restricted cytotoxic T-lymphocyte (CTL) epitopes, MHC class II-restricted helper T-lymphocyte (HTL) epitopes, and B-cell epitopes within a single construct is intended to simultaneously engage CD8+ cytotoxic T cells, CD4+ helper T cells (which in turn support antibody class-switching and the generation of long-lived memory), and antibody-producing B cells, a multi-arm strategy that more closely reproduces the breadth of a natural protective response than any single antigen or antibody-focused subunit vaccine can achieve, and one increasingly favored in rational vaccine design against genetically diverse pathogens [5,20]. It is worth being precise about what computational prediction can and cannot establish at this stage. Antigenicity scores and predicted immunogenicity describe the likelihood that a sequence will be recognized by the immune system and provoke a measurable response; protective immunity, the capacity of that response to actually prevent or clear infection, can only be established experimentally. The terms are related but not interchangeable, and the computational metrics reported in this study should be read as the former rather than as evidence of the latter. The case for anchoring epitope selection specifically in the conserved core genome follows from the same diversity that undermines conventional vaccines: because *S. suis* circulates as a large number of antigenically distinct serotypes and sequence types, an epitope repertoire drawn from variable, strain-restricted antigens is inherently vulnerable to immune escape through antigenic drift or the simple absence of the target protein in a non-vaccine-matched strain, whereas epitopes derived from genes conserved across the pan-genome are, by construction, less likely to be lost or diversified across the breadth of the circulating population.

Reverse vaccinology provides the methodological basis for this strategy. Since its introduction for the identification of vaccine candidates against serogroup B meningococcus directly from whole-genome sequence data, the approach has replaced culture-based antigen screening with computational mining of the genome for surface-exposed, essential, and non-host-homologous proteins [21]. The field has moved considerably further in the years since: structure-based epitope prediction and machine-learning and deep-learning models, including convolutional networks, transformers, and graph neural networks, now identify linear and conformational epitopes with substantially improved accuracy over sequence-based methods alone, and are beginning to surface antigenic targets that earlier, purely sequence-based pipelines would have missed [22]. Layered onto this is pangenomic analysis comparing gene content across many genomes of a single species to distinguish a conserved core genome from a variable accessory genome, which anchors antigen discovery in genes shared across the full breadth of a pathogen’s population rather than in any single reference strain [23,24]. This pangenome-guided variant of reverse vaccinology has recently been applied to other genetically diverse bacteria, where it has proven effective at surfacing conserved and previously unexploited antigenic targets [25]. A further, often underappreciated source of population-level failure in epitope-based vaccines is human MHC (HLA) polymorphism itself: because HLA allele frequencies vary substantially across ethnic groups, an epitope repertoire selected without explicit population-coverage analysis can inadvertently protect some populations far better than others, which is why population coverage against major HLA class I and class II alleles is treated here as a design criterion in its own right rather than an incidental output [26]. Because the construct designed in this study is intended for human immunization against a pathogen that is transmitted to people from an animal reservoir, epitope prediction and population-coverage analyses were carried out exclusively against human MHC class I and class II (HLA) alleles; evaluating binding to the MHC molecules of the non-human reservoir species would require a separate, species-specific epitope-prediction pipeline and falls outside the scope of the present, human-directed vaccine design [26].

Based on these considerations, the present study aimed to develop a rationally designed multi-epitope vaccine candidate against *S. suis* by integrating pangenome-guided reverse vaccinology with a comprehensive immunoinformatics framework. Unlike previous computational studies that relied primarily on a limited number of reported virulence factors or individual reference genomes [15,18,19], our strategy systematically prioritized conserved core proteins shared across multiple complete genomes through sequential pangenome analysis and subtractive proteomics, increasing the likelihood of identifying vaccine targets that are broadly conserved across the pathogen’s population rather than restricted to a small number of strains. The selected antigens were used for the prediction and rational assembly of cytotoxic T-lymphocyte, helper T-lymphocyte, and B-cell epitopes into a single chimeric construct, which was then evaluated *in silico* for antigenicity, safety, physicochemical properties, structural integrity, receptor-binding potential, molecular dynamics stability, simulated immune response, global HLA population coverage, and codon-optimized expression feasibility. None of these analyses can substitute for experimental immunology, and the value of this framework will ultimately depend on recombinant expression, *in vitro* confirmation of antigenicity and immunogenicity, and, most critically, immunization and pathogen-challenge studies in appropriate *S. suis* infection models. With that validation still to come, this work is best understood as a reproducible computational framework for prioritizing vaccine antigens in genetically diverse pathogens, a starting point rather than a finished candidate for subsequent experimental development.

## 2. Results

### 2.1. Pan-Genome Composition of S. suis

**Figure 1:**
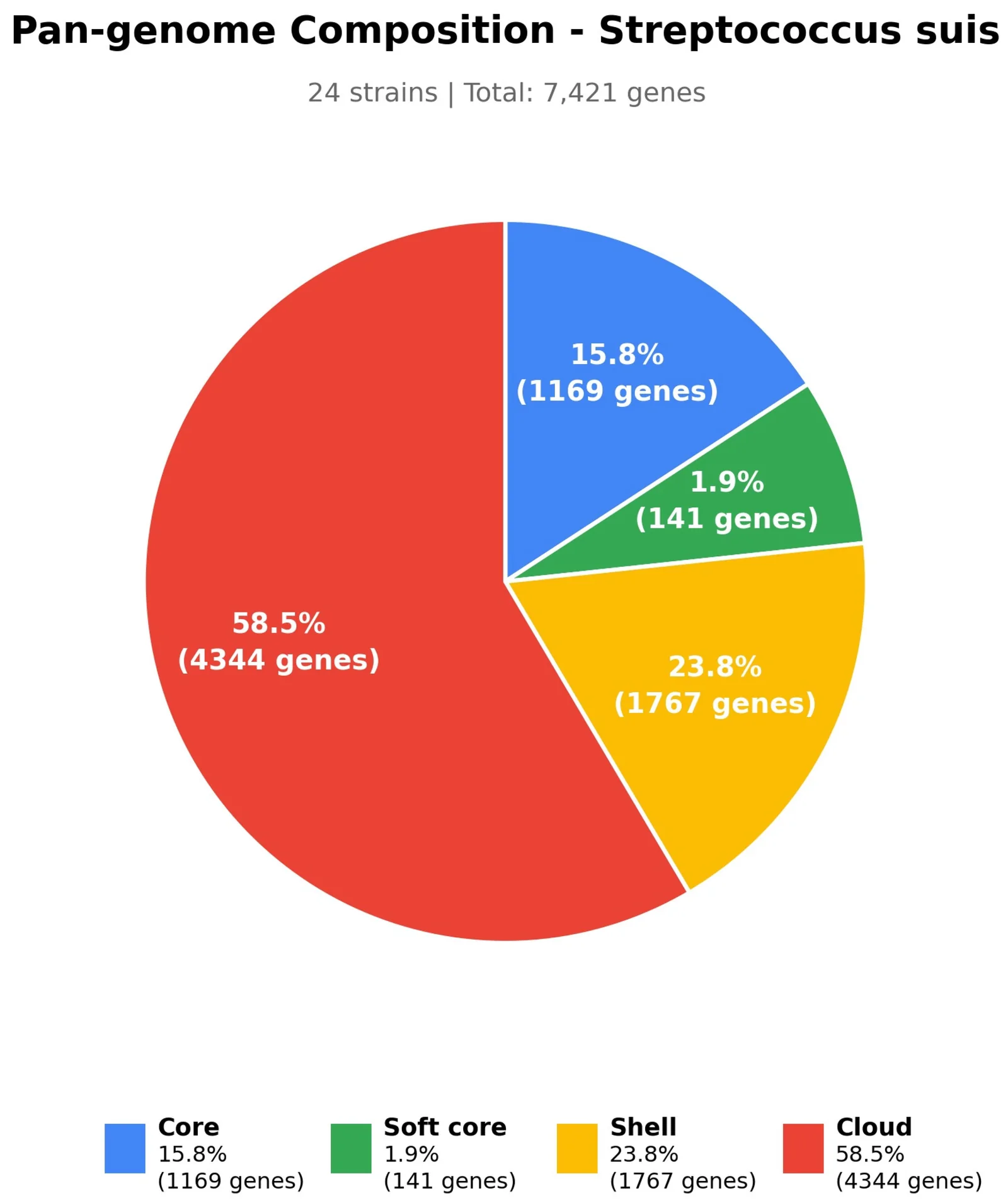
Twenty-four *S. suis* genomes were used to create this pan-genome with Panaroo. The pie chart shows the distribution of gene families into core (1169 genes, 15.8%), soft-core (141 genes, 1.9%), shell (1767 genes, 23.8%) and cloud (4344 genes, 58.5%) of the total pan-genome of 7421 gene families.

A total of 7421 gene families were found in the pan-genome of the twenty-four *S. suis* genomes and sorted into four categories based on the number of genome sequences in which the genes occurred (Figure 1). The core genome, which is the set of genes that are shared by at least 98% of the genomes analyzed, contained 15.8% (1169 genes). A small number of genes (141, 1.9%) were present in most strains but not in a few isolates and are termed the soft core genome. There were 1767 genes (23.8%) shared by several but not all strains that formed the shell genome. The cloud genome, consisting of 4344 genes (58.5%), represented the greatest share of the pan-genome, comprising genes that are strain-specific or rarely found among the isolates analyzed.

Cloud genes were more prevalent than core genes, suggesting that there is a large amount of genetic variation between the strains of *S. suis* analyzed and a small amount of variation in the core genome genes. The core genome was then extracted and translated into protein sequences to perform further subtractive proteomics and immunoinformatics analyses.

### 2.2. Subtractive Proteomics-Based Identification of Potential Vaccine Candidates

**Figure 2:**
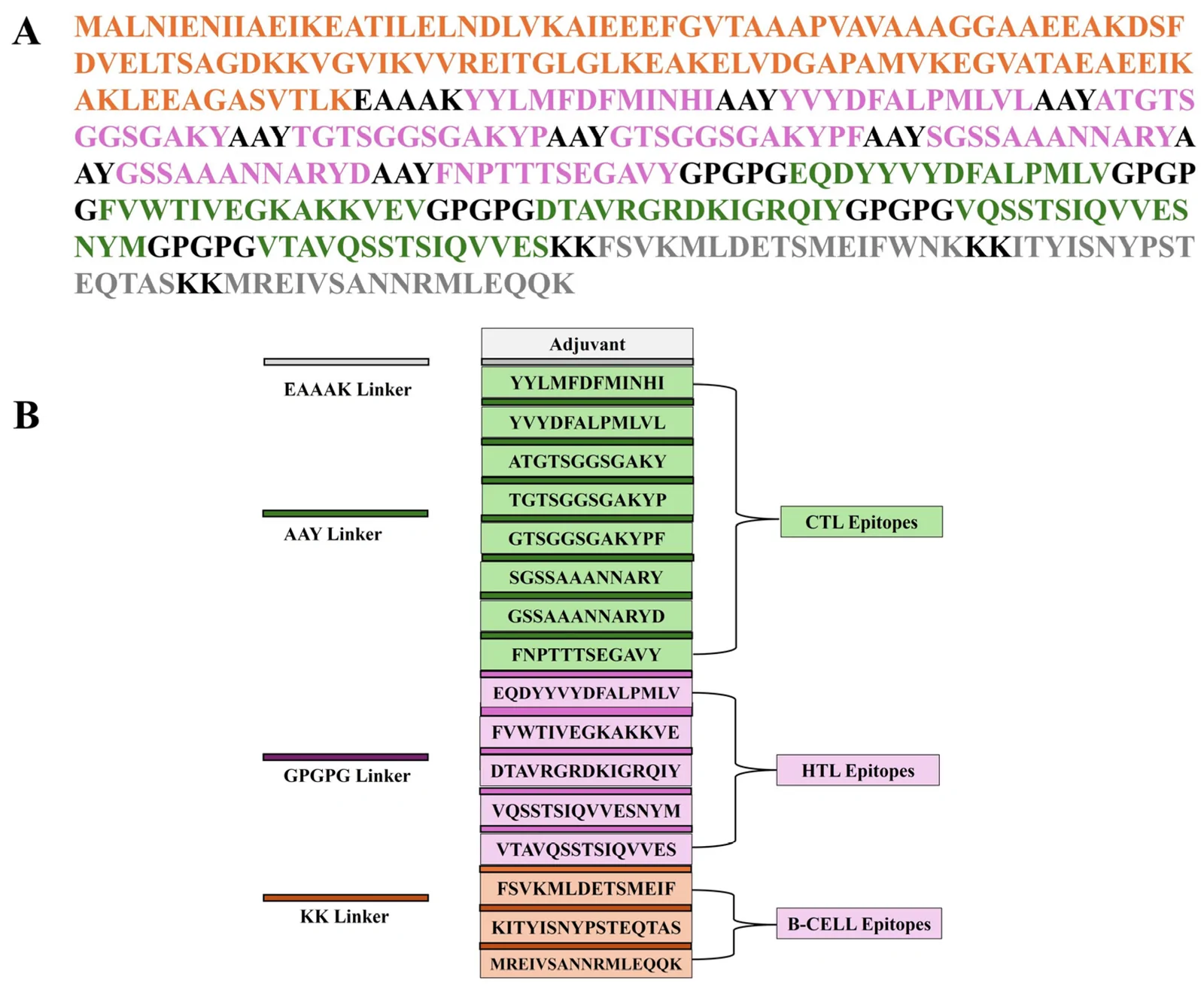
Design and organization of the multi-epitope vaccine (MEV) construct. (**A**) Complete amino acid sequence of the construct, showing the 50S ribosomal protein L7/L12 adjuvant in orange, cytotoxic T-lymphocyte (CTL) epitopes in purple, helper T-lymphocyte (HTL) epitopes in green, and linear B-cell epitopes in gray; linker sequences are shown in black. (**B**) Schematic organization of the MEV construct, comprising the adjuvant, eight CTL epitopes, five HTL epitopes, and three linear B-cell epitopes. The EAAAK, AAY, GPGPG, and KK linkers connect the adjuvant and the respective epitope groups. In panel B, CTL, HTL, and B-cell epitopes are shown in green, purple, and light orange, respectively.The non-homologous proteins were then checked against the Database of Essential Genes (DEG) to identify the essential proteins that are required for the survival of the bacteria. In this analysis, 187 essential proteins were identified. Physicochemical characterization of the filtered proteins was then performed based on their molecular weight, instability index and hydropathicity, and subcellular localization and transmembrane helices were predicted. The proteins that were present in the extracellular region or cell wall and had a single transmembrane helix or fewer were chosen for further studies. The antigenicity, allergenicity, and toxicity of these proteins were additionally studied, with the probability of adhesion being calculated.

A comprehensive subtractive proteomics pipeline was applied to the core proteome from the pangenome analysis to look for proteins present in the pathogen but not in the host, which have desirable vaccine characteristics (Figure 2). The translation of the core proteins was first compared with the human proteome to remove those showing high sequence similarity with host proteins. Of the 1169 core genes identified in the pangenome, nine did not yield valid complete protein sequences during extraction and translation, for example, partial open reading frames or sequences lacking an appropriate start codon; the remaining 1160 translated core proteins entered the subtractive proteomics pipeline. Of these 1160 core proteins, 365 were found to be non-homologous to the human proteome and used for further analysis, while the rest were excluded because they were homologous and highly similar to human proteins.

After sequential screening in the subtractive proteomics pipeline, three proteins met all of the previously set criteria and were identified as potential vaccine candidates: Sucrose phosphorylase (WP_174851906.1), an RND transporter-associated adaptor protein (WP_256768021.1), annotated in the source database as an efflux RND transporter periplasmic adaptor subunit, and Peptidoglycan hydrolase PcsB (WP_136628818.1) (Table 1). The selected protein WP_256768021.1, annotated in the source database as an efflux RND transporter periplasmic adaptor subunit, was further evaluated using independent computational localization and topology predictors because of the atypical localization expected for this annotation in *S. suis*. PSORTb did not assign a definitive localization and returned an overall prediction of “Unknown,” although a signal peptide was detected and equal localization scores were obtained for the cytoplasmic membrane, cell wall, and extracellular compartments. In contrast, CELLO predicted an extracellular localization as the most probable cellular compartment (score = 2.891), with a lower membrane-associated score (1.416). TMHMM predicted a single transmembrane helix spanning residues 9–31, followed by an extracellularly oriented region extending from residues 32–388. Collectively, these findings support a membrane-associated, non-cytoplasmic topology with a predominantly extracellular predicted domain. However, because the localization predictions were not completely concordant, the precise cellular localization of WP_256768021.1 remains computationally uncertain. The designation “periplasmic adaptor subunit” therefore reflects the source-database annotation and is not an experimentally established localization determined in the present study. The antigenicity scores of the selected proteins were 0.5927, 0.6414 and 0.6262, respectively, by the software VaxiJen v2.0. In addition, all three proteins were expected to be non-toxic and non-allergenic, facilitating their downstream epitope prediction and vaccine construction.

### 2.3. Prediction and Screening of Cytotoxic T Lymphocyte (CTL) Epitopes

The three proteins selected as candidate vaccines were analyzed for the presence of CTL epitopes using the IEDB MHC class I binding prediction tool. Predicted epitopes were then analyzed for their antigenicity, immunogenicity, allergenicity and toxicity properties. After sequential screening, eight CTL epitopes passed all the predefined selection criteria and were retained for further analysis (Table 2). The antigenicity scores of these epitopes were between 1.1096 and 2.9274, and they all had predicted binding affinity to several HLA class I alleles, suggesting the potential for recognition across multiple HLA class I backgrounds. Epitope selection was limited to the epitopes that were predicted to be antigenic and to lack allergenic and toxic properties.

### 2.4. Epitope Prediction and Evaluation of Helper T Lymphocyte (HTL) Epitope

The HTL epitopes were predicted using the IEDB MHC class II binding prediction tool and then further assessed for antigenicity, immunogenicity, allergenicity, toxicity and cytokine-inducing potential. Five HTL epitopes, meeting all the screening criteria, were selected for vaccine construction (Table 3). The chosen epitopes were found to bind multiple HLA-DR alleles, and their antigenicity scores were in the range 0.6784–1.3322, supporting their computational prioritization as candidate HLA class II-binding epitopes.

### 2.5. Prediction and Screening of Linear B-Cell Epitopes

The linear B-cell epitopes were predicted using the ABCpred server and then screened for antigenicity, immunogenicity, allergenicity and toxicity. Three B-cell epitopes were selected that satisfied all the previously defined selection criteria and were retained for insertion in the final vaccine construct (Table 4). The epitopes selected had ABCpred scores between 0.63 and 0.86 and antigenicity scores from 0.5027 to 0.7402. All the shortlisted epitopes were predicted to be non-allergenic and non-toxic, and thus suitable for the designed multi-epitope vaccine.

### 2.6. Conservation of Vaccine-Target Proteins

The selected CTL, HTL, and B-cell epitopes were derived from proteins identified within the conserved core proteome of the 24 analyzed *S. suis* genomes. Thus, the selection of epitope-containing proteins was based on their presence within the core protein set rather than on a separate residue-level conservation analysis of each individual epitope. This strategy ensured that the candidate epitopes originated from proteins consistently represented across the analyzed genomes. However, conservation of the parent protein does not necessarily establish complete conservation of every individual epitope sequence.

### 2.7. Multi-Epitope Vaccine (MEV) Construct

The resulting CTL, HTL and linear B-cell epitopes were selected and combined to form a final multi-epitope vaccine (MEV). The 50S ribosomal protein L7/L12 was added as an adjuvant to the N-terminal end of the construct and coupled to the first CTL epitope via an EAAAK linker to increase its immunogenicity. The individual CTL epitopes were linked with AAY linkers; the HTL epitopes were linked with GPGPG, while the linear B-cell epitopes were separated by KK for efficient antigen processing and presentation. The final vaccine construct was a 397-amino-acid construct containing one adjuvant, eight CTL epitopes, five HTL epitopes and three linear B-cell epitopes, all linked together in sequence. The schematic organization and full amino acid sequence of the designed MEV are shown in Figure 2.

### 2.8. Predicted Population Coverage of the Selected T-Cell Epitopes

**Figure 3:**
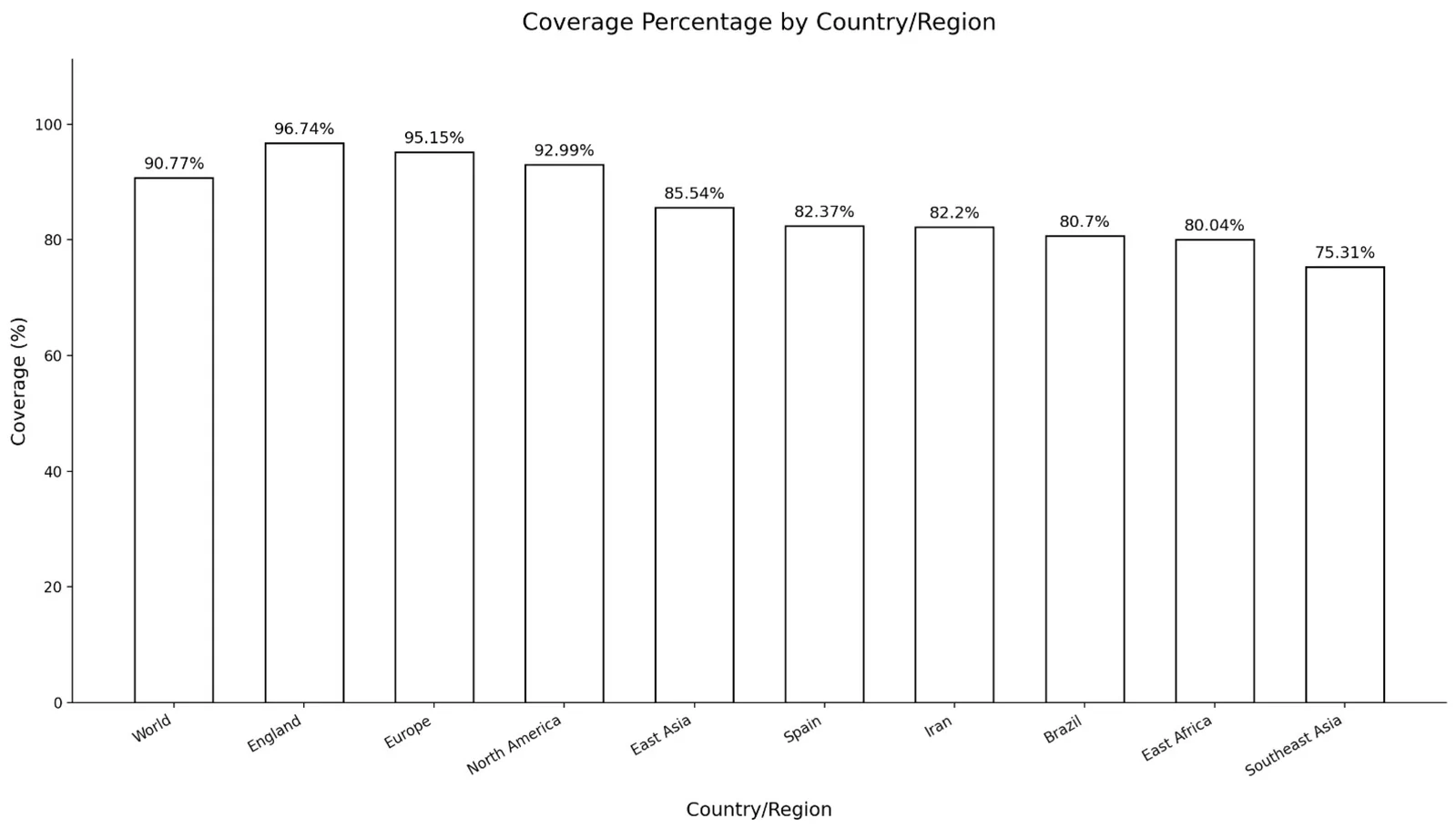
The extent of global and regional population coverage of the selected CTL and HTL epitopes by the corresponding HLA alleles. The combined predicted HLA population coverage across the evaluated populations was 90.77%.

Selected CTL and HTL epitopes together with the corresponding class I and class II HLA alleles were analyzed to calculate theoretical population coverage in various geographical regions. The predicted combined HLA population coverage was 90.77% across the populations included in the analysis (Figure 3). The results of the regional analysis revealed good population coverage at different geographic locations. Coverage was greatest in England (96.74%), Europe (95.15%) and North America (92.99%). East Asia (85.54%), Spain (82.37%), Iran (82.20%), Brazil (80.70%) and East Africa (80.04%) showed high coverage, while Southeast Asia showed the lowest predicted coverage (75.31%) among the evaluated regions. These estimates indicate broad predicted HLA representation of the selected CTL and HTL epitopes across the evaluated populations; they should not be interpreted as predicted vaccine efficacy or protective coverage.

### 2.9. Immunological and Physicochemical Characterization of the Multi-Epitope Vaccine Construct

Immunological and physicochemical properties of the designed multi-epitope vaccine (MEV) construct were assessed for their suitability for use in vaccine development work. The final build consisted of 397 amino acids, with a molecular weight of 41.58 kDa and an isoelectric point (pI) of 5.15, which is slightly acidic. The amino acid composition showed that alanine (15.4%) was the most abundant residue and glycine (10.8%), valine (8.1%), glutamic acid (7.8%), lysine (7.3%) and serine (7.3%) were the other important ones. The construct had 44 negative and 37 positive amino acid residues.

The Instability Index calculated by ProtParam analysis was 34.81, which indicates that the construct was expected to be a stable protein. The aliphatic index was 76.07, indicating that the thermostability of the protein is good, and the GRAVY value of −0.128 indicated the overall hydrophilic nature of the protein. The half-life of the construct was estimated to be 30 h in mammalian reticulocytes (*in vitro*), >20 h in yeast and >10 h in *Escherichia coli*, which suggests good stability across expression systems. Further, the extinction coefficient was predicted to be 46,760 M^−1^cm^−1^, which is useful for efficient spectrophotometric detection in protein purification and characterization. These physicochemical properties indicate that the designed MEV construct is suitable for further structural and immunological investigations.

### 2.10. Secondary Structure Prediction and Three-Dimensional Structure Validation

The secondary structure of the designed MEV construct was predicted with the SOPMA server. The model predicted 156 α-helices (39.29%), 68 extended β-strands (17.13%) and 173 random coils (43.58%), showing an even distribution of ordered and flexible structural elements. Structured and flexible areas are present, indicating an architecture conducive to epitope presentation and immune recognition.

**Figure 4:**
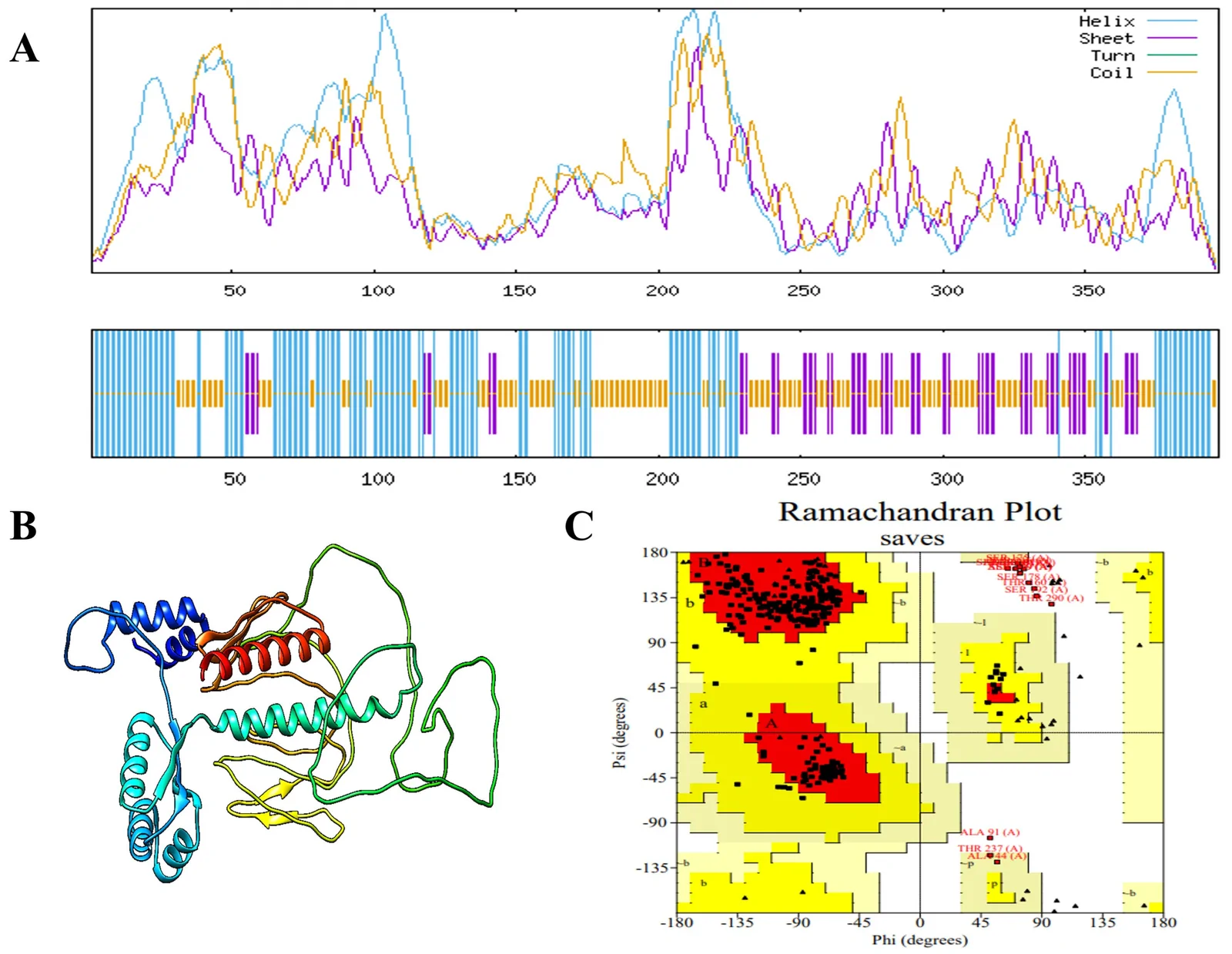
Structural characterization of the designed multi-epitope vaccine construct. (**A**) Predicted secondary-structure composition and residue-wise distribution, with α-helices shown in light blue, β-sheets in purple, turns in green, and coils in orange. (**B**) Refined three-dimensional model of the vaccine construct shown using a rainbow color gradient from the N-terminus in blue to the C-terminus in red; helices, β-strands, and loops are represented by spirals, arrows, and connecting coils, respectively. (**C**) Ramachandran plot showing the stereochemical quality of the refined model. Red, yellow, pale-yellow, and white regions represent the most favored, additionally allowed, generously allowed, and disallowed conformational regions, respectively. Squares represent non-glycine residues, triangles represent glycine residues, letters identify the corresponding conformational regions, and red labels indicate residues located outside the allowed regions.

The 3D structure of the vaccine construct was then predicted and optimized to ensure that vaccine stability was maintained, and structural validation was carried out by implementing various quality assessment tools. The stereochemical quality of the refined model was assessed by Ramachandran plot analysis, with 89.2% of residues in the most favored, 6.6% in the additionally allowed, 0.6% in the generously allowed and only 3.6% in the disallowed regions (Figure 4).

**Figure 5:**
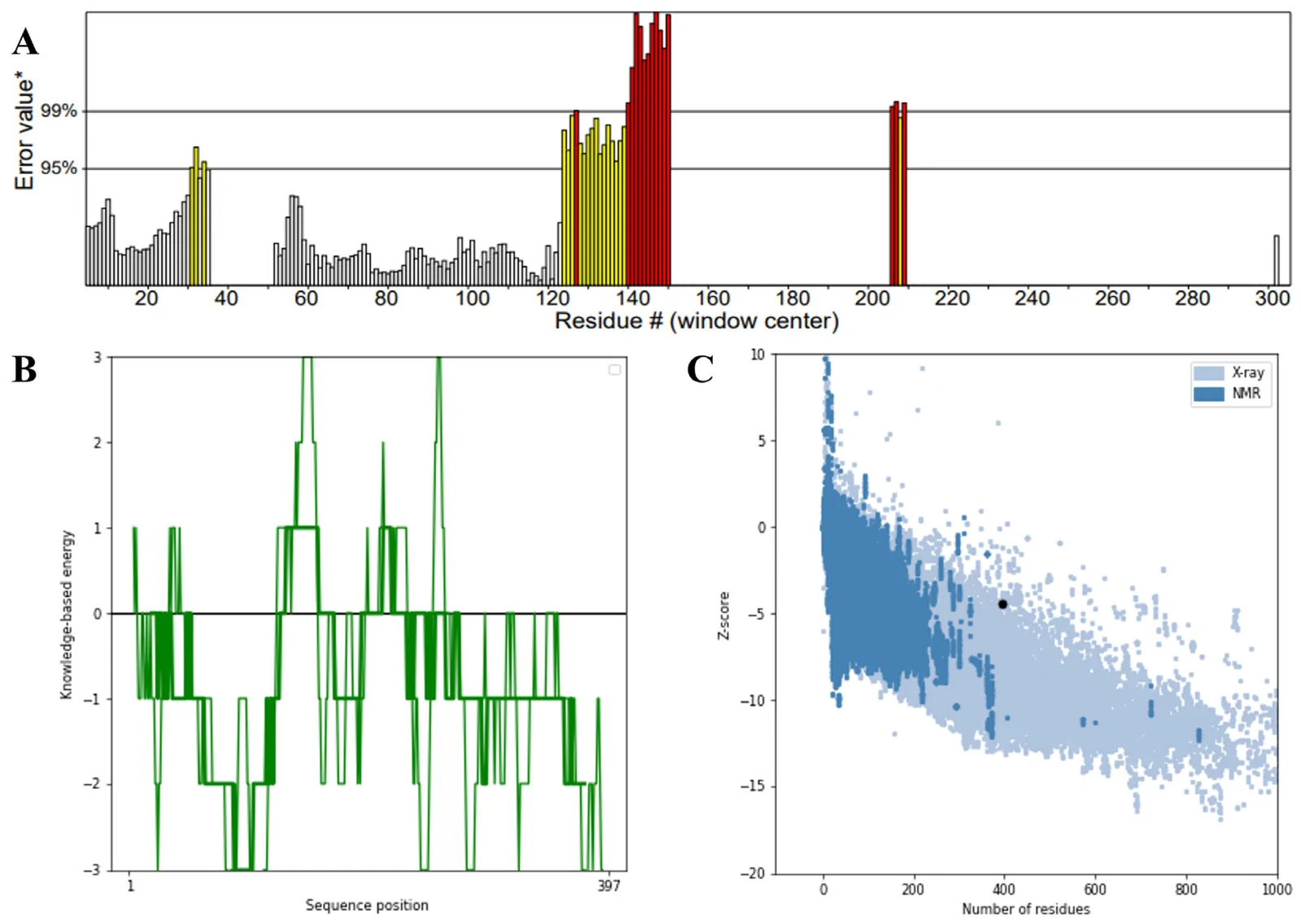
Validation of the predicted three-dimensional structure of the multi-epitope vaccine construct. (**A**) ERRAT analysis showing residue-wise error values based on non-bonded atomic interactions. The asterisk (*) refers to the ERRAT error value, while the number sign (#) denotes the residue number. White bars indicate regions below the 95% rejection threshold, yellow bars indicate regions between the 95% and 99% thresholds, and red bars indicate regions exceeding the 99% rejection threshold. (**B**) ProSA-web local energy profile showing the knowledge-based energy across the vaccine sequence. (**C**) ProSA-web Z-score plot comparing the modeled vaccine construct with experimentally determined protein structures. Light-blue and dark-blue points represent structures determined by X-ray crystallography and NMR spectroscopy, respectively, while the black point represents the modeled vaccine construct.

The predicted tertiary structure was further validated with the ERRAT server, which gave an overall quality factor of 79.67, indicating the reliability of the predicted structure. The ProSA-web analysis resulted in a Z-score of −4.43, characteristic of the range of typically good protein structures determined experimentally for proteins of similar size (Figure 5). These secondary structure prediction and structural validation analyses indicate that the designed MEV construct has a stable 3D structure and is appropriate for conformational epitope prediction, molecular docking and molecular dynamics simulation in the next step.

### 2.11. Conformational B-Cell Epitope Prediction

To identify both linear and conformational B-cell epitopes, the designed multi-epitope vaccine (MEV) construct was analyzed by the ElliPro server that provides the refined three-dimensional structure. ElliPro identified 6 linear B-cell epitopes between 9 and 98 amino acids in length and predicted that the scores of these epitopes were in the range of 0.586 to 0.799. The best linear epitope comprised 98 amino acid residues (positions 152–249) with a prediction score of 0.799, while there was a prediction score range of 0.586–0.753 for the other epitopes.

**Figure 6:**
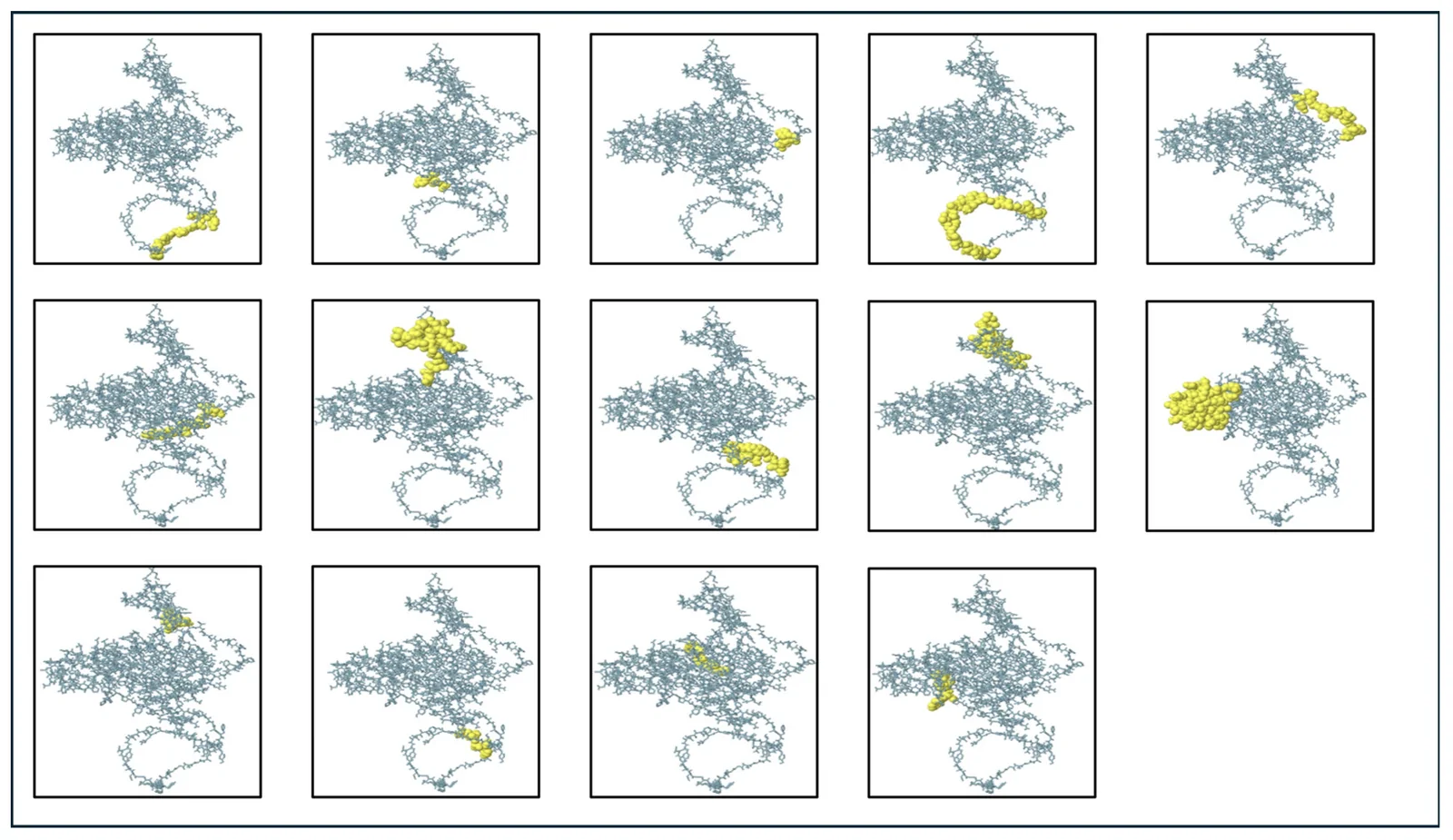
Discontinuous (conformational) B-cell epitopes of the designed multi-epitope vaccine construct were predicted using predicted antigen-binding regions. Fourteen discontinuous B-cell epitopes were identified in the refined 3D structure of the vaccine construct by the ElliPro analysis. The complete vaccine construct is shown in blue-gray, whereas the residues forming each predicted discontinuous B-cell epitope are highlighted in yellow. The predicted epitopes are spread throughout the surface-exposed region, suggesting that there are multiple antigenic sites that may be accessible to antibodies and thus potentially able to elicit humoral immune responses.

Furthermore, the locations of the 14 discontinuous (conformational) B-cell epitopes were determined by the spatial arrangement of the amino acid residues in the tertiary structure after refinement. The predicted conformational epitopes were 3 to 47 residues in length and had ElliPro scores between 0.519 and 0.956. The 14-residue conformational epitope (positions 183–196) that was most highly predicted (a score of 0.956) was followed by the 3-residue (a score of 0.909) and 4-residue (a score of 0.902) epitopes. Multiple surface-exposed antigenic regions are also predicted throughout the vaccine construct, including several larger conformational epitopes of 26, 28 and 47 residues. The predicted linear and conformational B-cell epitopes indicate that the designed MEV construct has several accessible antigenic regions that have the potential to generate humoral immune responses (Figure 6).

### 2.12. Molecular Docking of the Multi-Epitope Vaccine Construct

Protein–protein docking was performed between the designed multi-epitope vaccine (MEV) construct and the TLR1/TLR2 heterodimer (PDB ID: 2Z7X) using the ClusPro 2.0 protein–protein docking server. Of the docking models generated, Cluster 0 with 59 members was found to be the most reliable cluster. The representative model from this cluster had a weighted docking score of −1037.1, and the lowest energy score of the cluster was −1277.4, suggesting a good binding conformation between the vaccine construct and the receptor.

The docked complex was further analyzed with the PDBsum server, where Chain A is considered the receptor and Chain S is the vaccine construct. The hydrogen bonds, salt bridges, and non-bonded contacts observed in the interface analysis showed that there is a predicted network of intermolecular contacts between the interacting proteins, with a total of 33 hydrogen bonds, 3 salt bridges, and 78 non-bonded contacts. The number of hydrogen bonds, electrostatic and hydrophobic interactions indicates that the docked complex is consistent with a plausible modeled interaction interface.

**Figure 7:**
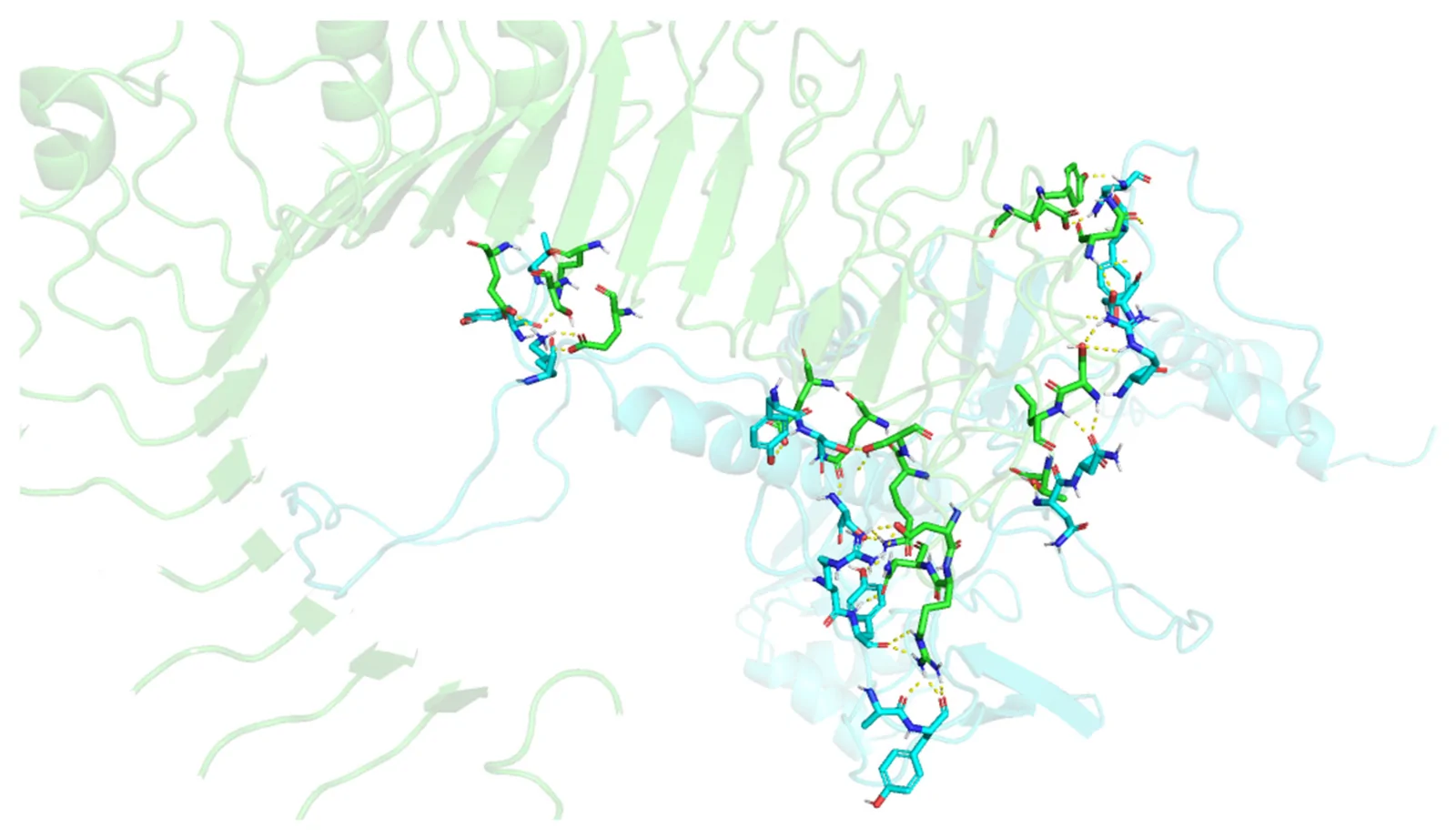
The molecular docking and molecular interaction analysis of the multi-epitope vaccine construct with the TLR1/TLR2 receptor (PDB ID: 2Z7X). The TLR1/TLR2 receptor (Chain A) and vaccine construct (Chain S) are shown in pale green and cyan, respectively. Interfacial residues are displayed as sticks, with oxygen and nitrogen atoms shown in red and blue, respectively; yellow dashed lines indicate predicted hydrogen-bonding interactions. PDBsum analysis identified 33 hydrogen bonds, 3 salt bridges, and 78 non-bonded contacts at the interface. PRODIGY predicted a binding free energy (ΔG) of −16.9 kcal mol^−1^ and an equilibrium dissociation constant (K_d) of 3.8 × 10^−13^ M.

The docked complex was then assessed for its binding affinity, and the thermodynamic properties were calculated using the PRODIGY server. The predicted binding free energy (ΔG) was found to be −16.9 kcal mol^−1^ and the estimated equilibrium dissociation constant (Kd) was 3.8 × 10^−13^ M, a favorable protein–protein interaction energy and a low estimated dissociation constant; however, these values represent computational predictions and do not establish experimentally confirmed receptor binding or biological activity. Overall, the docking, interaction and binding analyses support the formation of a stable receptor–vaccine complex, which is suitable for further molecular dynamics simulation (Figure 7).

### 2.13. Normal Mode Analysis of Docked Complex

**Figure 8:**
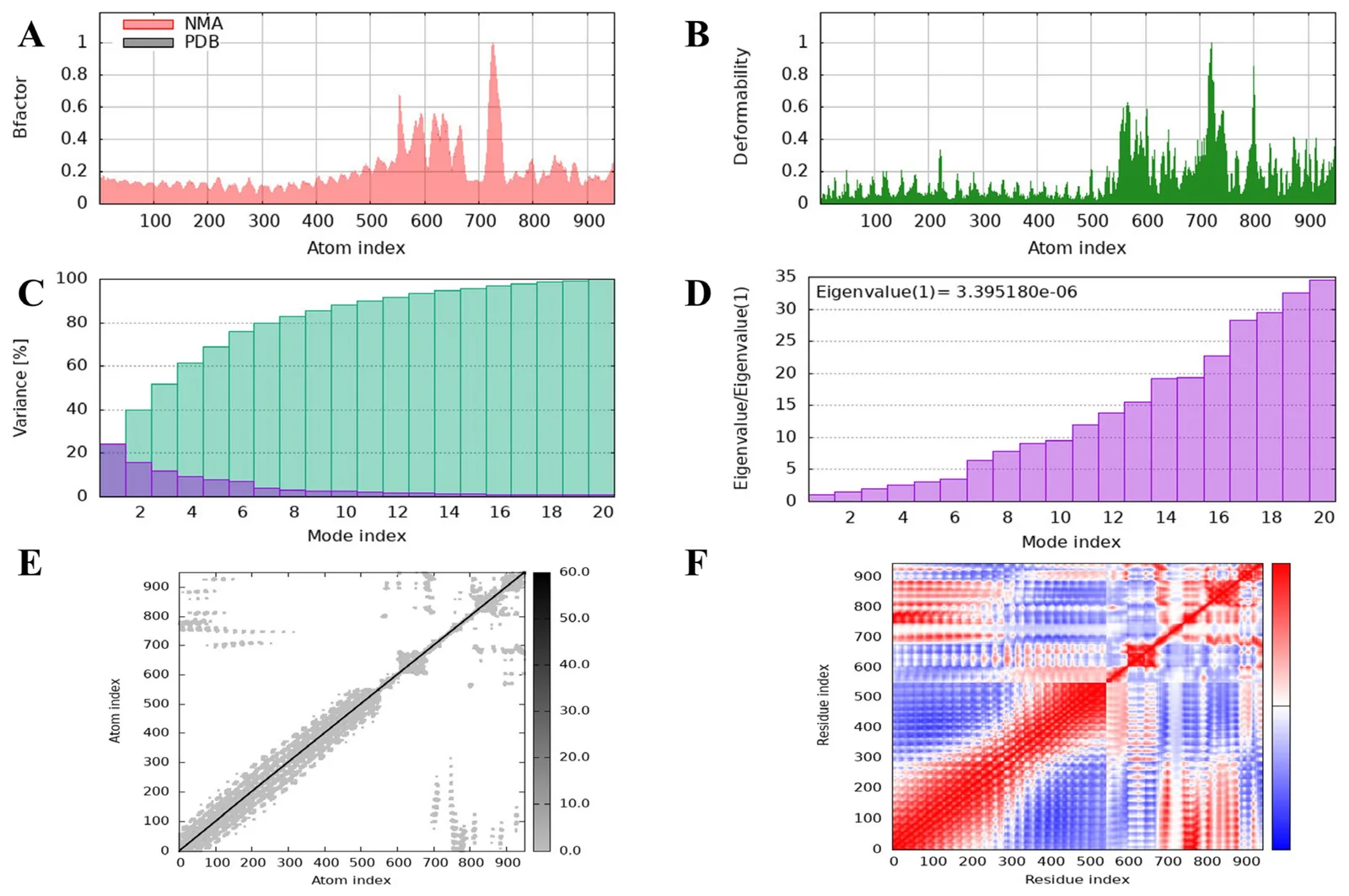
Normal mode analysis (NMA) of the vaccine–receptor complex. (**A**) B-factor profiles, where pink represents the NMA-predicted values and gray represents the PDB-derived values. (**B**) Deformability plot, in which the green peaks indicate regions with relatively high predicted flexibility. (**C**) Variance plot, where purple represents the variance contributed by each individual mode and green represents the cumulative variance. (**D**) Eigenvalue distribution across the normal modes; the software-generated notation 3.395180 × 10^−6^ is equivalent to 3.395180 × 10^−6^. (**E**) Elastic-network map, in which gray-to-black lines represent connections between atom pairs, with darker lines indicating stiffer interactions; the prominent diagonal reflects strong local connections between neighboring atoms. (**F**) Covariance matrix, where red, blue, and white indicate correlated, anticorrelated, and uncorrelated atomic motions, respectively.

The structural stability and intrinsic flexibility of the docked vaccine receptor complex were investigated by a Normal Mode Analysis (NMA) performed via the iMODS server. The predicted B-factor profile was in good agreement with the experimentally obtained one, except for a few regions where the flexibility was relatively higher. The B-factors for most of the residues showed low values, suggesting that the structure of the complex was stable, with the flexible loop regions (residues 550–750) displaying higher B-factors that could enable conformational changes in the binding process of molecular recognition (Figure 8A).

Based on the deformability analysis, the majority of the residues showed minimal deformation, while a few localized peaks were seen, mainly in the region of residues 550–800, indicating the existence of flexible hinge regions that might be able to undergo conformational changes without significantly impacting the overall structure of the complex (Figure 8B). For the complex, energy was calculated and found to be 3.39518 × 10^−6^, which means that only a small amount of energy is needed for conformational changes. This small eigenvalue shows high collective mobility and robust structural stability for the vaccine receptor complex, which indicates that it can undergo motions that are biologically relevant and necessary for effective molecular interactions (Figure 8C).

The variance analysis showed that the variance was growing with each subsequent “normal” mode (or normal frequency), implying that a major portion of the total variance in the collective motions in the complex occurs in the lower-frequency modes. This suggests that low-frequency normal modes are of fundamental importance in large-scale functional movements (Figure 8D). The motions of the residues in the complex were correlated and anti-correlated, as shown by the covariance matrix. The positively correlated movements are found along the diagonal; the negatively correlated regions are found to represent coordinated domain motion between the various parts of the complex. These collective motions help maintain the overall stability of the vaccine–receptor complex, but at the same time, offer significant flexibility for the complex to adjust its structure (Figure 8E).

In addition, the elastic network model showed that the complex was tightly coupled and had a dense network of inter-residue interactions throughout it, suggesting that there is a high degree of mechanical coupling between the residues. The connectivity observed in the elastic network is high, indicating that there is a high degree of structural rigidity which enables the integrity of the complex to be maintained whilst allowing for local flexibility to enable receptor binding (Figure 8F). In general, the NMA results show that the vaccine receptor complex has a good balance of structure and flexibility. On this basis, the docked complex was carried forward to molecular dynamics simulation.

### 2.14. Molecular Dynamics Simulation Analysis

**Figure 9:**
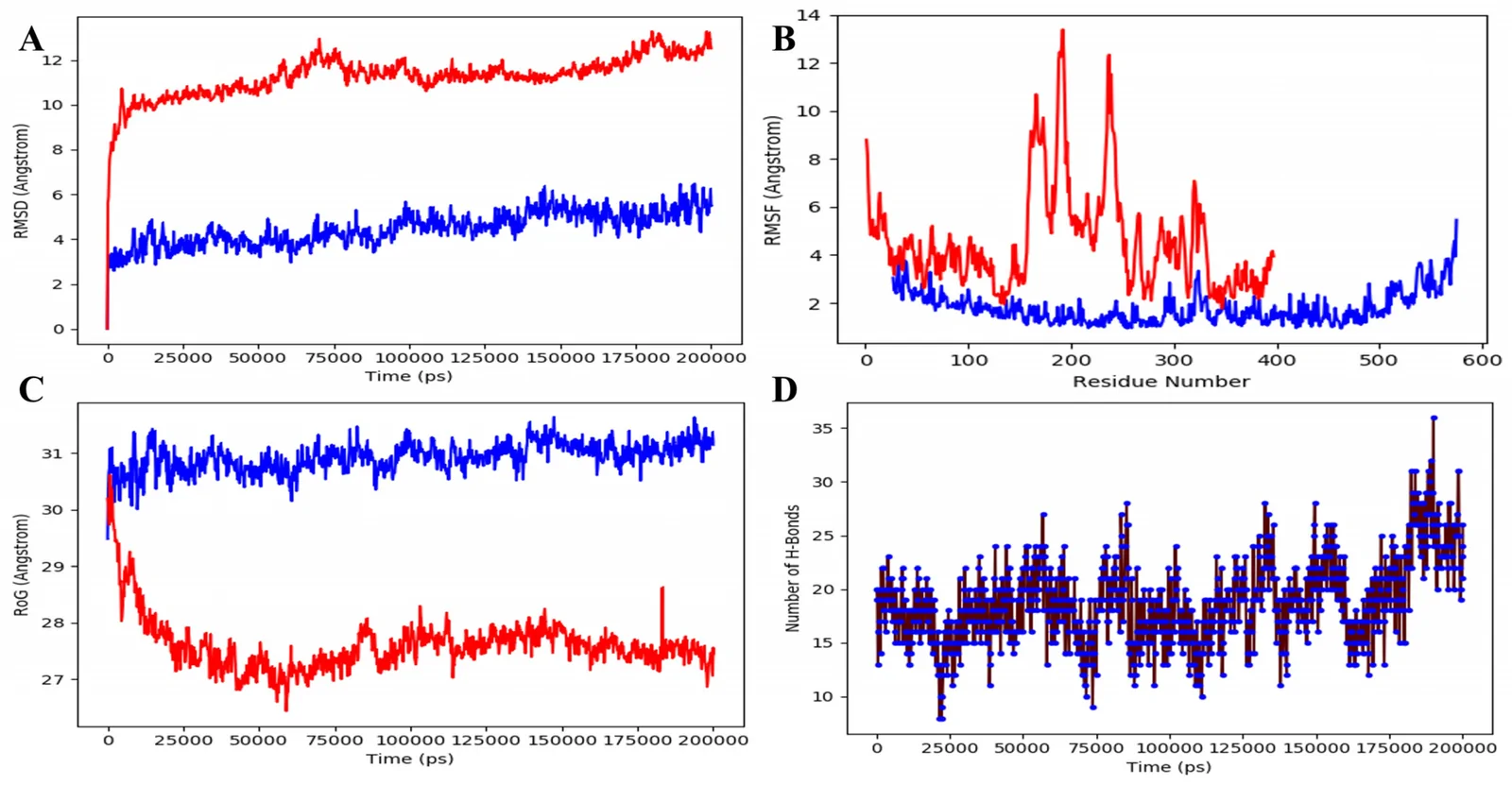
Over the course of the 200 ns molecular dynamics simulation, the vaccine–receptor complex was analyzed. The blue curves represent the receptor, with the red curves being the vaccine construct. (**A**) Root mean square deviation (RMSD) for structural stability of the receptor and vaccine construct during the simulation. In (**B**), root mean square fluctuation (RMSF) is shown, which represents flexibility at the residue level. The compactness of the molecules is shown by (**C**) Radius of gyration (Rg) throughout the simulation. (**D**) Number of intermolecular hydrogen bonds (H-bonds) between the vaccine construct and receptor during the simulation trajectory; blue markers show the calculated hydrogen-bond counts at individual simulation frames, while the dark-red connecting lines illustrate their temporal variation.

The structural stability and dynamic behavior of the vaccine–receptor complex were evaluated by performing a molecular dynamics (MD) simulation of 200 ns duration. Root mean square deviation (RMSD), root mean square fluctuation (RMSF), radius of gyration (Rg) and hydrogen bond analyses were used to assess the stability of the individual components (Figure 9).

The RMSD analysis showed that the receptor (blue) and the vaccine construct (red) quickly reached equilibrium in the early portion of the simulation (Figure 9A). The receptor showed relatively minor structural deviations during the simulation, with RMSD values ranging from around 3.0 Å to 6.0 Å, which indicates limited conformational drift within the analyzed trajectory. The vaccine construct exhibited a comparatively high RMSD of approximately 10–13 Å after the initial equilibration phase. However, the absence of a sustained upward trend suggests that the construct reached a relatively stable region of conformational sampling rather than continuing to undergo progressive structural drift.

The RMSF profile showed that the receptor has relatively low values compared to the RMSF of the residues of other proteins; most of the residues had RMSF values of less than 3 Å (Figure 9B), suggesting that the receptor has relatively low fluctuation at the residue level. The vaccine construct, on the other hand, exhibited greater flexibility, especially in several loops, where the conformational changes were about 13 Å. The localized peaks are likely due to flexible linker or epitope regions, while the rest of the residues showed relatively small fluctuations, indicating the general stability of the construct.

The radius of gyration (Rg) analysis revealed the two molecules to have different structural characteristics (Figure 9C). It was found that the Rg value of the receptor remained almost unchanged during the simulation, with the average value being around 30–31 Å, indicating that the compact tertiary structure of the receptor is preserved. The vaccine construct, in contrast, showed initial compaction with Rg dropping to values of approximately 27–28 Å, which were then maintained during the early part of the simulation, followed by more ordered folded structures.

Intermolecular interactions were found throughout the whole simulation by hydrogen bond analysis (Figure 9D). The number of H-bonds ranged from about 8 to 36, with an average of ~18–25 H-bonds maintained throughout most of the simulation. The hydrogen bond interactions gradually increased towards the end of the trajectory, indicating the stabilization of the vaccine-receptor interaction with the passage of time.

Overall, the MD analysis indicated that the vaccine construct sampled a broader conformational space than the receptor, and that both components settled into sustained conformational states over the 200 ns trajectory. Neither the RMSD profile nor the residue-level fluctuations showed progressive drift, and intermolecular hydrogen bonding was maintained throughout. These observations describe the conformational behavior of the modeled complex within the simulated timeframe and should not be interpreted as experimental evidence of structural stability.

### 2.15. Principal Component Analysis (PCA)

**Figure 10:**
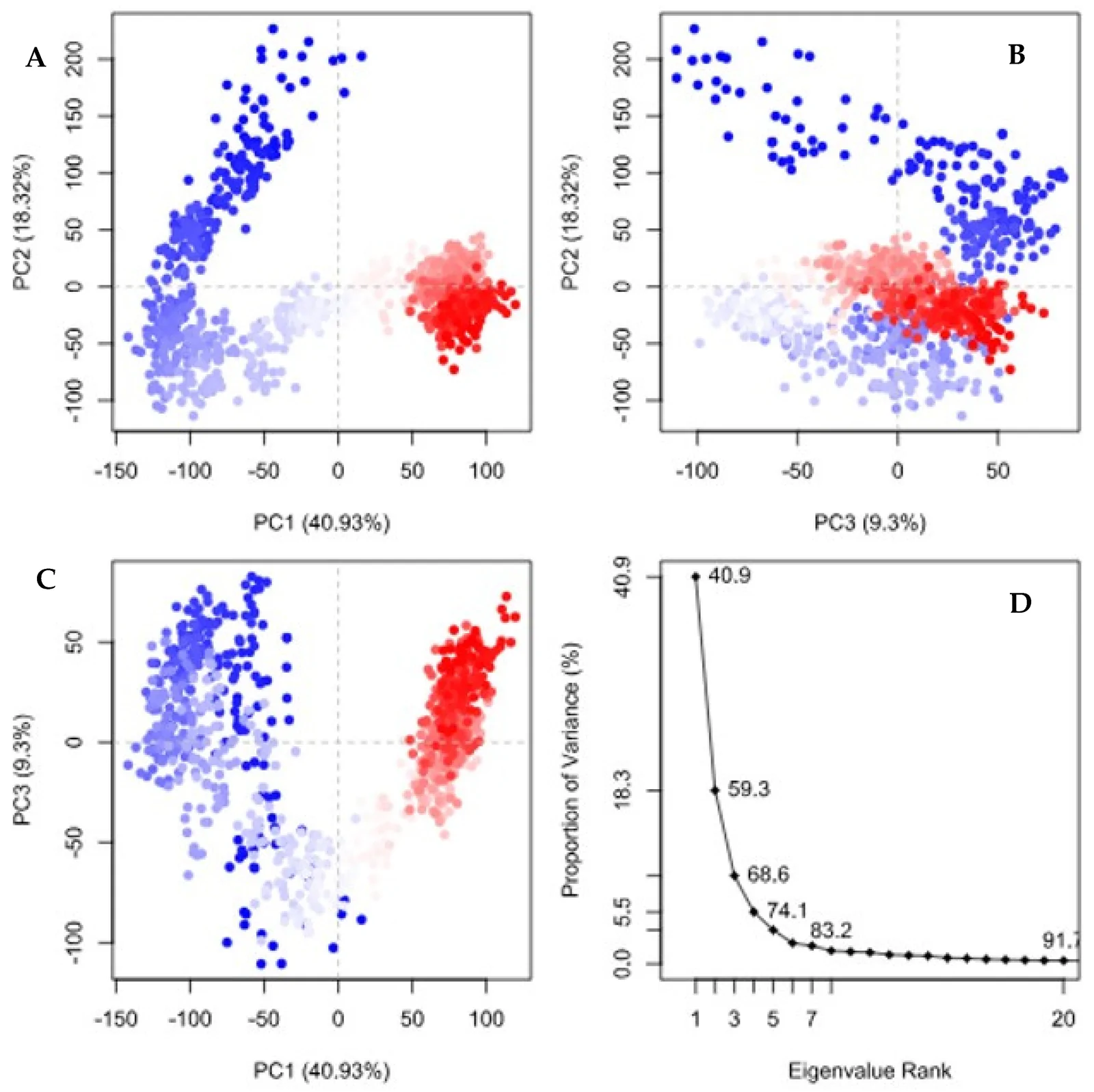
The principal component analyses of the vaccine–receptor complex, projecting onto the PCs: (**A**) PC1 vs. PC2, (**B**) PC2 vs. PC3, (**C**) PC1 vs. PC3 projections, and (**D**) the scree plot, which shows the variance explained by each principal component. In panels (**A**–**C**), the blue-to-red color gradient represents the progression of conformational states sampled during the simulation trajectory, from earlier frames in blue to later frames in red. In panel (**D**), the bars show the percentage of variance explained by successive principal components.

The dominant collective motions of the vaccine–receptor complex were characterized by performing 200 ns molecular dynamics simulation of the complex using principal component analysis (PCA) (Figure 10). Conformational projections along the first three principal components (PC1, PC2, PC3) showed that the conformational space sampled during the simulation was quite distinct. The scatter plots showed that there were clearly identifiable conformational clusters with gradual changes between conformations, which indicated that the complex sampled several distinguishable conformational states within the analyzed trajectory (Figure 10A–C).

The first principal component (PC1) explained 40.93% of the total variance, while the second principal component (PC2) and third principal component (PC3) explained 18.32% and 9.30% of the total variance, respectively. The first three principal components accounted for about 68.6% of the total number of atomic motions, meaning that most of the important dynamics of the complex were represented by these low-frequency motions (Figure 10D).

When the simulation trajectory was plotted in the subspaces defined by the top three principal components, PC1–PC2, PC2–PC3, and PC1–PC3, conformational changes were observed but not sudden. The distribution of conformations indicated that the complex sampled a bounded region of conformational space over the course of the simulation, combining limited overall displacement with appreciable local flexibility.

The scree plot also revealed that the contribution of the next few principal components was quite small, suggesting that the variance decreases drastically after the first few principal components, which contributed most to the overall variance in molecular motions. The cumulative variance rose to 40.9% for PC1, 59.3% for PC2, 68.6% for PC3, 74.1% for PC4 and 83.2% for PC7, and finally to about 91.1% for the first twenty principal components. These results suggest that the essential motions of the vaccine–receptor complex are dominated by a few low-frequency collective modes.

The PCA projections indicated that the complex sampled several conformational states during the 200 ns trajectory, with the dominant collective motions captured by the first few principal components and the dispersion in the PC projections remaining bounded. These observations characterize the conformational behavior of the modeled complex within the simulated timeframe but should not be interpreted as experimental evidence of structural stability.

### 2.16. MM-GBSA Binding Free Energy Analysis

MM-GBSA analysis was performed to further assess the predicted binding energetics of the vaccine construct–TLR complex. The calculated binding free-energy values were −340.91 and −266.96 kcal mol^−1^, with a mean predicted ΔG_bind of −303.93 ± 52.29 kcal mol^−1^ (SD, *n* = 2). The favorable negative values indicate energetically favorable association of the modeled vaccine–receptor complex within the applied MM-GBSA framework. These values represent computational estimates of binding energetics and should not be interpreted as experimentally measured binding affinities or direct evidence of immunological activity.

### 2.17. Immune Simulation Analysis

**Figure 11:**
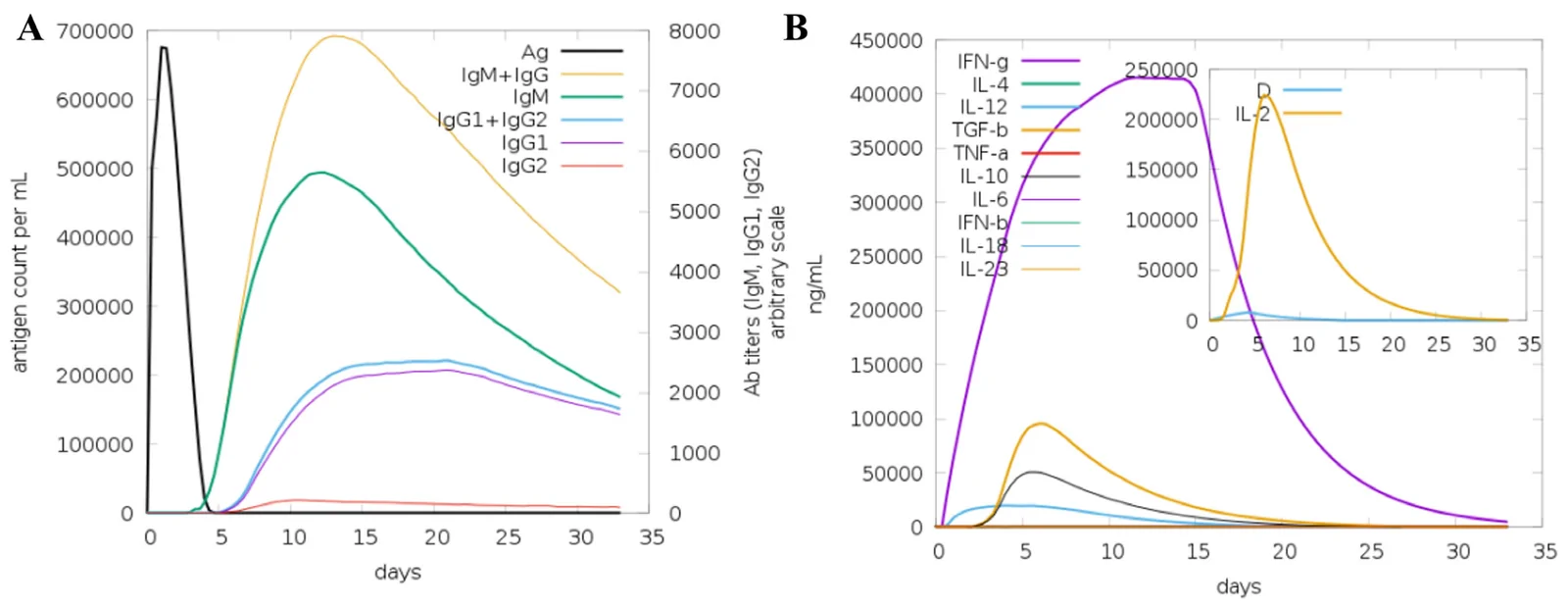
Immune simulation results showing (**A**) antigen clearance and antibody responses following vaccination and (**B**) predicted cytokine profiles following vaccination.

The immune simulation showed that the immune response (humoral and cellular) against the designed multi-epitope vaccine was strong and coordinated (Figure 11). The antigen concentration rose quickly from day 0 to 2, and peaked prior to a rapid decrease, suggesting effective antigen recognition and clearance by the host immune system (Figure 11A).

There was a marked antibody response following antigen exposure. The overall antibody level (IgM + IgG) rose rapidly, reaching maximum levels around day 14 and then gradually decreasing while maintaining a high level throughout the simulation period. The first immune response was mostly an IgM antibody response, while the secondary antibody response was an IgG1 and IgG2 antibody response. The IgG1 + IgG2 response persisted at a high level post-peak, consistent with predicted IgG class switching and simulated humoral memory. IgG2, however, showed lower levels compared to IgG1, suggesting that the IgG1-mediated response was predominant (Figure 11A).

The cytokine profile also showed a strong activation of the immune system after vaccination (Figure 11B). Of all the cytokines, interferon-γ (IFN-γ) showed the highest expression level, peaking around days 14–16, indicating a robust Th1 response with a cell-mediated immune response. Increased levels of IL-2 were also detected during early phases of the immune response, thus promoting T cell proliferation and activation. An increase in the levels of IL-10, IL-12 and IL-6 was seen, which shows the balanced regulation of both pro-inflammatory and immunomodulatory pathways. In contrast, the levels of the cytokines TNF-α, TGF-β, IFN-β, IL-4, IL-18, and IL-23 were relatively low throughout the simulation, indicating a controlled inflammatory response with immune activation.

Immune simulation results overall indicate that the proposed multi-epitope vaccine was predicted to generate an antigen-specific humoral response with long-lasting antibody production and a well-balanced immune response from T cells, with a noticeable Th1-biased response. These findings suggest the potential for persistent immune responses that require experimental validation because of the efficient clearance of antigens, high antibody titers and high production of both IFN-γ and IL-2.

### 2.18. Codon Optimization and In Silico Cloning Results

The multi-epitope vaccine construct was successfully codon-optimized for *Escherichia coli* (strain K12) using the Java Codon Adaptation Tool (JCat). After codon optimization, the codon adaptation index (CAI) value was 0.996, which shows very good codon usage compatibility with the translational machinery of *E. coli*. The optimized sequence had a GC content of 48.78%, within the optimum range of 30–70%, indicating good stability of mRNA and translation efficiency.

The vaccine construct was successfully cloned *in silico* into the pET-30a(+) expression vector using the Benchling molecular biology platform, after codon optimization. The optimized gene was directionally cloned into the multiple cloning site (MCS) of the vector with the restriction enzyme sites EcoRI and HindIII, maintaining the reading frame to allow the expression of recombinant proteins.

**Figure 12:**
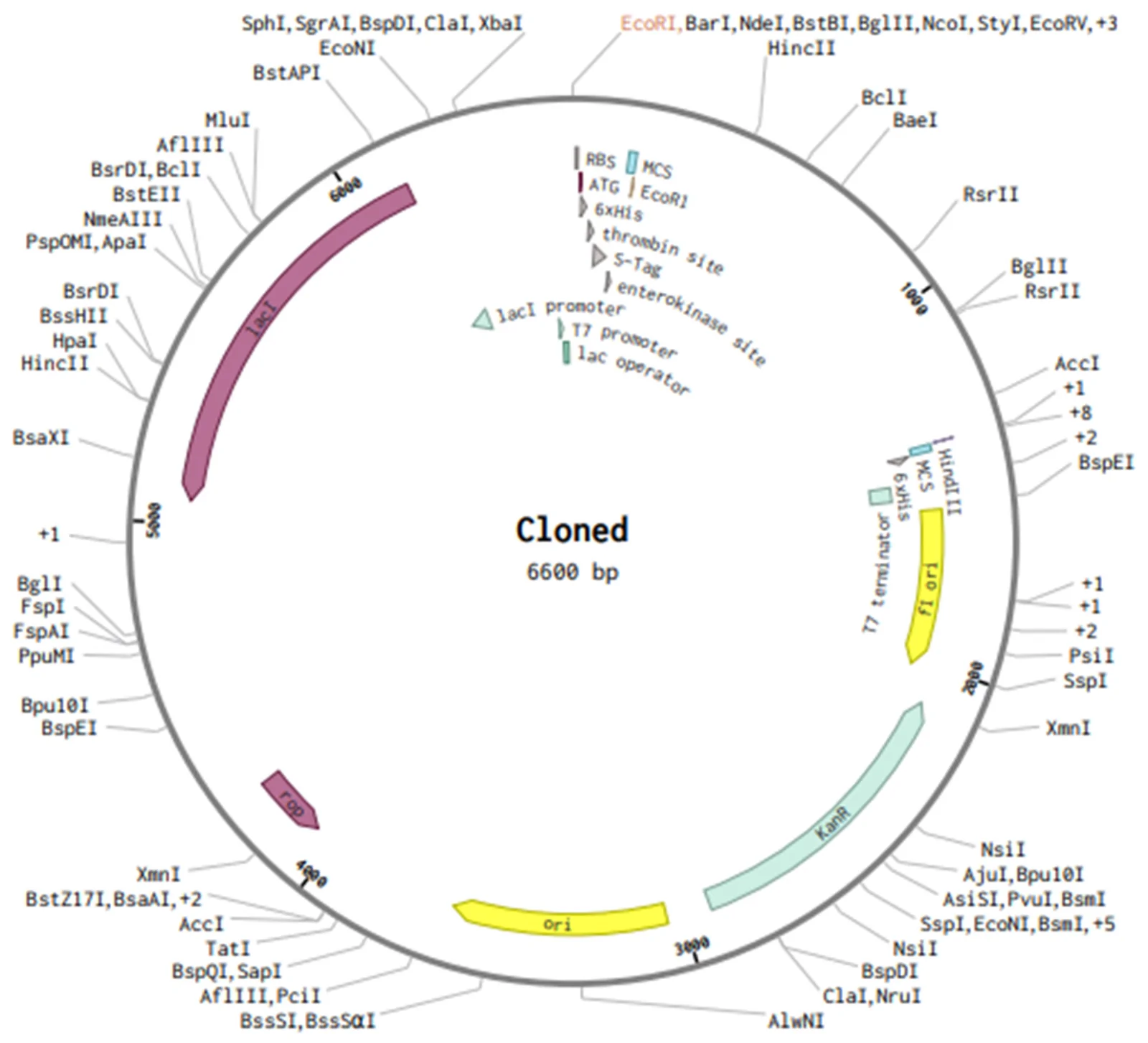
*In silico* cloning of the codon-optimized multi-epitope vaccine sequence into the pET-30a(+) expression vector using the Benchling web platform. The insert was positioned between the EcoRI and HindIII restriction sites, producing a predicted recombinant plasmid of 6600 bp. The gray circular line represents the plasmid backbone, the colored arrows indicate annotated functional elements and their orientations, and the surrounding labels identify restriction-enzyme sites. Colors correspond to the different feature categories automatically assigned by Benchling.

The T7 promoter and ribosome-binding site (RBS) were placed upstream of the recombinant construct, while the N-terminal 6 × His tag of the vector remained intact to allow for easy protein purification later. The final recombinant plasmid was expected to be 6600 bp, which is the vector backbone of pET-30a(+) along with the inserted vaccine construct. *In silico* analysis confirmed the expected orientation and reading frame of the insert and indicated the potential suitability of the construct for subsequent experimental expression in *E. coli*. The entire *in silico* cloning strategy is shown in Figure 12.

## 3. Discussion

The steady rise of antimicrobial resistance (AMR) is reshaping how we think about infectious disease, and not in a reassuring way. Drug-resistant infections already claim hundreds of thousands of lives each year, and the trajectory is worsening; some estimates suggest the annual toll could reach millions if the pipeline of effective countermeasures continues to lag behind the pace of resistance evolution [3,6]. *S. suis* sits uncomfortably at the intersection of this crisis and the growing threat of zoonotic disease. For decades it was treated as a veterinary concern, a pathogen of pigs with little direct relevance to human medicine, but that view has not aged well. *S. suis* is now recognized as a genuine human pathogen capable of causing meningitis, septicemia, endocarditis, and arthritis, and its reach extends well beyond farm workers and slaughterhouse employees to broader communities in regions where people and livestock share close living environments [7,8]. Much of what makes it so difficult to manage clinically comes down to the combination of traits it brings: more than 35 serotypes, a demonstrated ability to form biofilms, and resistance profiles that have crept upward across multiple antibiotic classes, steadily narrowing the options available to clinicians [12,13]. Developing a new generation of vaccines against *S. suis* is therefore not a scientific exercise so much as a practical necessity. Multi-epitope vaccine (MEV) design, guided by reverse vaccinology and immunoinformatics, offers a principled route forward, making it possible to identify conserved antigenic targets across serotypes and assemble them into constructs engineered to provoke broad, durable protective immunity [27,28].

To move beyond the limitations of strain-specific and literature-driven target selection, we adopted a pangenome-driven reverse vaccinology strategy aimed at candidates conserved across diverse *S. suis* strains. Pangenome analysis of 24 complete, quality-filtered genomes resolved a total of 7421 gene families, comprising a core genome of 1169 genes (15.8%), a soft-core genome of 141 genes (1.9%), a shell genome of 1767 genes (23.8%), and a cloud genome of 4344 genes (58.5%). The predominance of cloud genes indicates substantial genomic diversity among *S. suis* strains, whereas the conserved core genome represents a stable genetic repertoire shared across nearly all isolates. The core genome, encoding the fundamental machinery required for bacterial survival and fitness, formed the basis of our antigen screening pipeline. This mirrors established pangenome frameworks in streptococcal species, where the core genome captures conserved physiological functions while the accessory genome encodes niche-specific adaptations and virulence traits [23,24]. A recent pangenome analysis of *S. suis* isolates from Hubei Province, China, identified 943 core genes among 19 strains spanning ten serotypes, reinforcing the view that the core genome of this pathogen, though compact, remains a reliable reservoir of broadly conserved targets [29]. Antigens encoded in the core genome are strategically attractive because they are unlikely to be lost through horizontal gene transfer or subject to serotype-specific antigenic variation, precisely what a broadly protective vaccine requires. One caveat applies: Heaps’ law analysis and formal pangenome saturation assessment were not performed here, so the long-term stability of the observed core genome fraction as additional genomes are added cannot be fully established. Larger and more geographically diverse *S. suis* genome collections will be needed to resolve pangenome openness and core genome dynamics, and to firm up confidence in the targets identified.

Our subtractive proteomics pipeline, filtering sequentially for essentiality, absence of human homology, and predicted surface or extracellular accessibility, identified three candidate antigens: sucrose phosphorylase (WP_174851906.1), peptidoglycan hydrolase PcsB (WP_136628818.1), and an RND transporter-associated adaptor protein (WP_256768021.1), annotated in the source database as an efflux RND transporter periplasmic adaptor subunit. Excluding proteins with human homologs by BlastP (E-value ≤ 10^−5^; sequence identity < 30%) is a well-established safeguard against cross-reactive autoimmune responses [30]. PSORTb version 3.0.3 and CELLO v.2.5 localization analysis predicted extracellular or surface-exposed positioning for all three, and essentiality predictions confirmed that each is required for bacterial viability, two features that together define high-value targets under the reverse vaccinology framework [21,31]. Computational localization carries inherent uncertainty, however, and direct experimental verification of surface exposure in *S. suis* remains an important next step. A 2024 study on *Streptococcus gordonii* using a near-identical pangenome–subtractive proteomics workflow likewise converged on surface-accessible, non-homologous core proteins as its leading MEV candidates [32], which lends methodological support to the route we took.

The three proteins each bring a distinct and complementary rationale to the design. Sucrose phosphorylase (WP_174851906.1) is a glycosyltransferase-family enzyme that catalyzes the reversible phosphorolysis of sucrose into glucose-1-phosphate and fructose, placing it at a metabolic junction central to bacterial energy acquisition and carbohydrate homeostasis. Carbohydrate-metabolizing enzymes, phosphorylases involved in polysaccharide precursor synthesis among them, have been linked to biofilm accumulation and host colonization in streptococci, with intracellular polysaccharide stores serving as an endogenous energy reservoir that sustains persistence under nutrient-limited conditions [33,34]. Given its extracellular localization and essentiality, antibody-mediated disruption of this enzyme could plausibly impair metabolic fitness and colonization at the same time. Peptidoglycan hydrolase PcsB (WP_136628818.1) is a CHAP domain-containing murein hydrolase that coordinates peptidoglycan remodeling during cell division, mediating septum splitting and daughter-cell separation in streptococci. Its functional coupling to the membrane-spanning FtsEX ATPase complex has been worked out mechanistically in *Streptococcus pneumoniae*, where PcsB is essential across all capsule serotypes and its depletion produces pronounced chaining, aberrant morphology, and growth arrest [35]. A 2025 study combining biochemical and structural approaches provided the most detailed model yet of how ATP hydrolysis in FtsE is transduced through FtsX to activate PcsB during septal splitting, underscoring how indispensable it is to the division cycle [36]. Broad conservation across streptococcal species, surface localization, and essentiality together make PcsB one of the most compelling targets we identified. The third candidate, WP_256768021.1, is annotated in the source database as an efflux RND transporter periplasmic adaptor subunit. Proteins carrying this annotation belong to the resistance-nodulation-division (RND) family of tripartite transporters, which rank among the most clinically significant drivers of multidrug resistance in bacteria [37,38], and in which the periplasmic adaptor bridges the inner-membrane transporter and the outer-membrane channel. Whether that architecture applies here is not established by the present analyses, which support only a predicted membrane-associated, non-cytoplasmic topology of uncertain precise localization. The protein was therefore prioritized on the basis of the subtractive proteomics criteria it satisfied, and its localization, functional role, and any contribution to efflux-mediated resistance require experimental characterization. Efflux-pump components have nonetheless been proposed as vaccine antigens in other organisms [39].

From the three prioritized antigens, we predicted high-affinity CTL, HTL, and linear B-cell epitopes using established immunoinformatics tools and assembled them into a single MEV construct designed to engage coordinated cellular and humoral immunity. Combining MHC class I-restricted CTL epitopes, MHC class II-restricted HTL epitopes, and B-cell epitopes recruits CD8^+^ cytotoxic T cells, CD4^+^ helper T cells, and antibody-producing B cells simultaneously, a multi-arm strategy increasingly favored over single-component approaches because it more closely reproduces the breadth of a natural protective response [20]. Population coverage analysis predicted a combined HLA population coverage of 90.77% across the populations evaluated, comparable with values reported for other published streptococcal MEV designs [18,19]. No single construct is likely to achieve complete universal coverage given the extreme polymorphism of HLA alleles across human populations, but this breadth indicates broad predicted HLA representation across the populations evaluated rather than demonstrated protective coverage. The final epitope set was deliberately kept compact to reduce immunodominance competition and limit junctional neo-epitope formation, considerations now standard in rigorous MEV design [26,40].

The construct, spanning 397 amino acids, was assembled by joining the selected epitopes through four established linkers: EAAAK, AAY, GPGPG, and KK. These spacers physically separate adjacent epitopes, reduce steric clashes, and preserve the conformational integrity needed for immune recognition, a design logic validated extensively across published MEV studies [41]. *S. suis* 50S ribosomal protein L7/L12 was incorporated as an N-terminal adjuvant to drive innate priming through predicted interaction with the Toll-like receptor (TLR1/TLR2), coupling innate sensing to adaptive activation. ProtParam analysis predicted a molecular weight of approximately 41.58 kDa, an instability index of 34.81, and favorable solubility, all consistent with feasibility for recombinant expression. Dedicated linker-junction neo-epitope screening was not undertaken here; the linkers selected do, however, have an established record of minimizing junctional immunogenicity in comparable constructs [41]. Even so, future designs would benefit from explicit computational neo-epitope screening at junction sites to further reduce the risk of unwanted immune diversion.

Structural modeling and iterative refinement confirmed a stable, well-organized tertiary architecture. Ramachandran analysis placed 89.2% of residues in favored conformational regions, with disallowed residues confined to flexible loop and linker segments, regions expected to adopt non-canonical backbone geometries without compromising functional or immunogenic core elements. ProSA-web returned a Z-score of −4.43, within the range of experimentally resolved structures of comparable molecular weight [42]. These metrics echo those of recent high-quality MEV studies, where thorough structural assessment is now treated as an obligatory checkpoint before docking and simulation [43,44]. Rigorous as it is, computational structure prediction remains an approximation; X-ray crystallography or cryo-electron microscopy will ultimately be required to confirm the folded architecture of the expressed construct.

Molecular docking predicted a favorable interaction between the refined MEV and TLR1/TLR2; the representative model from Cluster 0 had a ClusPro weighted score of 1037.1, consistent with favorable receptor engagement. TLR1/TLR2 was prioritized as the docking target because the L7/L12 adjuvant, together with bacterial surface-derived peptide epitopes, has been documented to activate TLR1/TLR2–NF-κB signaling, promoting pro-inflammatory cytokine production and enhanced antigen presentation [45,46,47]. Molecular dynamics (MD) simulations over 200 nanoseconds characterized the conformational behavior of the MEV–TLR1/TLR2 complex under the simulated conditions: the receptor deviated only modestly, the vaccine construct sampled a wider conformational range without progressive drift, the radius of gyration remained within a narrow band, and intermolecular hydrogen bonding persisted across the trajectory [48,49]. These are computational observations within the analyzed trajectory and do not establish experimental structural stability. Encouraging as these results are, *in silico* docking cannot by itself confirm receptor activation. Co-immunoprecipitation, surface plasmon resonance, or cell-based TLR1/TLR2 reporter assays will be needed for that, and future work should extend the analysis to TLR2, TLR1/2, and NOD-like receptors for a fuller picture of innate receptor engagement.

Computational immune simulation predicted robust, sustained adaptive responses to MEV administration, including high-titer IgG, persistent IFN-γ and IL-2 secretion, and the generation of long-lived memory B and T cell populations. These profiles align closely with predictions for other rationally designed MEVs and support the premise that the construct could induce durable immunity [50]. Codon optimization for expression in *Escherichia coli* yielded a CAI of 0.996 (optimal range 0.8–1.0) and a GC content of 48.78%, both within windows associated with efficient heterologous production [51]. *In silico* cloning into the pET-30a(+) vector confirmed translational feasibility and provides a practical starting point for recombinant production. These expression parameters are, of course, predictions; actual yield and solubility of the expressed MEV will have to be determined empirically during development.

The construct holds up well against existing *S. suis* MEV studies. Liang et al. [18] built a multi-epitope candidate from immunoinformatics—predicted epitopes of conserved virulence-associated surface proteins—reporting strong predicted antigenicity and sound structural quality, a solid foundation, but one that stopped short of experimental confirmation. Liu et al. [19] went considerably further in a 2024 npj Vaccines study, engineering three multi-epitope antigens from conserved proteins spanning 11 prevalent *S. suis* serotypes and testing them in a mouse model. The results were striking: robust humoral and cellular responses, meaningful cross-serotype protection, and positive opsonophagocytic activity. That work matters to us beyond its own findings, because it shows that computationally identified conserved epitopes can in fact function in a living system, lending biological credibility to the immunoinformatics-driven approach we followed. The present study differs primarily in the specific integration of pangenome-guided antigen prioritization with sequential subtractive proteomics and downstream immunoinformatics analyses. This should be considered a computational prioritization strategy rather than a fundamentally new vaccine-design methodology. What sets the present study apart is that our three targets (sucrose phosphorylase, PcsB, and the RND transporter-associated adaptor protein) were not drawn from the virulence literature but emerged from a systematic pangenome-guided screen of the core genome, giving greater confidence that they are genuinely conserved across the full breadth of *S. suis* genomic diversity. The RND transporter-associated adaptor protein deserves particular mention: to our knowledge, it has not previously been proposed as an *S. suis* vaccine target, although its localization, functional role, and suitability as an antigen remain to be established experimentally [39].

The significance of these findings extends past *S. suis* itself. Reverse vaccinology and immunoinformatics have matured over the past several years from largely exploratory exercises into a design pipeline that has produced a large and growing body of MEV candidates [52,53]. A meaningful number of these have now been carried through to *in vitro* and *in vivo* testing rather than left as computational proposals, reporting antigenicity, antibody induction, and protective immunity in animal models that track the predictions originally made for them. The cross-serotype protection Sanduja et al. achieved in mice [54] is the most directly relevant streptococcal example. This accumulating evidence lends empirical grounding to the assumptions behind our pipeline, without lessening the need for target-specific confirmation: immunogenicity demonstrated for one construct cannot be assumed for another, and direct immunization and challenge studies remain the only reliable test of whether a given candidate performs as designed.

This study provides a comprehensive and methodologically rigorous computational foundation for *S. suis* MEV development, but several limitations need to be stated plainly. The analysis is entirely *in silico*, and computational predictions, however well validated, are not a substitute for experimental evidence; the structural, immunogenic, and functional properties of the construct must be confirmed in the laboratory and in animals before any claim about efficacy can be made. Docking here was restricted to TLR1/TLR2, and future work should extend docking and simulation to TLR2, TLR4, TLR6, NOD1, and NOD2 to map the full spectrum of innate receptor interactions. A further limitation is that epitope conservation was inferred from the conservation of their parent proteins within the core proteome rather than being directly evaluated at the amino acid level for each selected epitope. Therefore, additional residue-level comparative analysis across a larger and more geographically diverse collection of *S. suis* genomes will be required to confirm the exact sequence conservation and broader applicability of the selected epitopes. The MD simulation timeframe and the number of independent replicates may also need to be extended to achieve statistical robustness in stability assessments. Dedicated linker-junction neo-epitope screening should be built into future design iterations to minimize the risk of unintended immunological diversion. Post-translational modifications, beyond the reach of current computational tools, may further modulate the immunogenic and structural behavior of the expressed protein. Experimental follow-up should prioritize recombinant expression and purification, antigenicity confirmation by ELISA and Western blot, dendritic cell activation assays, cytokine profiling, and lymphocyte proliferation analysis. The decisive test, though, will be animal immunization and challenge in appropriate *S. suis* infection models, assessing protective efficacy, safety, and the breadth and durability of the response, the indispensable bridge between what we have predicted here and what might one day protect both animals and people from this pathogen [43,44,55].

## 4. Materials and Methods

### 4.1. Genome Acquisition, Annotation, and Pangenome Construction

Twenty-four complete genome sequences of *S. suis* were obtained from the National Center for Biotechnology Information (NCBI) GenBank online database to perform a pangenome analysis [56]. Assemblies were chosen for their completeness, the quality of their annotation, and their suitability for comparative genomic studies. The accession numbers of all genomes included in this study are provided in Supplementary Table S1.

The genomic annotation (GFF3) and corresponding nucleotide sequence (FNA) files were downloaded for each genome. The annotation files were preprocessed to create Panaroo-compatible GFF3 files with a custom Python 3 script before the pangenome analysis. In the pre-processing step, the nucleotide sequences were added to the annotation file; the FASTA description lines were standardized; inconsistencies in the sequence identifiers were corrected; and the invalid formatting that would interfere with downstream analysis was removed.

Pangenome analysis was carried out using a bacterial genome pangenome analysis pipeline called Panaroo (version 1.3) [57]. Twenty-four genomes were used as the input for the prepared GFF3 files. The execution of Panaroo employed the moderate cleaning mode to fix inconsistencies in the annotations, and the -remove-invalid-genes option was used to remove fragments of genes and incomplete genes at the same time. Core gene alignment was generated by MAFFT version 7 [58], and core genes were defined as genes found in the genomes of 98% of the analyzed genomes, with the core threshold set at 0.98. Eight computational threads were used in the analysis, with the following parameters: --clean-mode moderate, --remove-invalid-genes, -alignment core, -aligner mafft, -core_threshold 0.98 and -t 8.

The resulting pangenome was segmented into 4 groups based on the distribution of genes among all the genomes analyzed in this study, namely core genes, soft-core genes, shell genes and cloud genes. The core genome identified by Panaroo was extracted and all subsequent subtractive proteomics and immunoinformatics analyses were performed on it [45,46,47].

Molecular dynamics (MD) simulations over 200 nanoseconds characterized the conformational behavior of the MEV–TLR1/TLR2 complex under the simulated conditions: the receptor deviated only modestly, the vaccine construct sampled a wider conformational range without progressive drift, the radius of gyration remained within a narrow band, and intermolecular hydrogen bonding persisted across the trajectory [48,49]. These are computational observations within the analyzed trajectory and do not establish experimental structural stability. Encouraging as these results are, in silico docking cannot by itself confirm receptor activation. Co-immunoprecipitation, surface plasmon resonance, or cell-based TLR1/TLR2 reporter assays will be needed for that, and future work should extend the analysis to TLR2, TLR1/2, and NOD-like receptors for a fuller picture of innate receptor engagement.

### 4.2. Core Gene Extraction and Protein Sequence Derivation

After the pangenome construction, the core gene clusters obtained by Panaroo were collected from the gene presence–absence matrix. The list of conserved core genes was used to identify the nucleotide sequences in the annotated genome assemblies. Each of the core genes was next translated to amino acid sequences using a custom Python script with implementation of the bacterial genetic code translation Table 11, the Bacterial, Archaeal and Plant Plastid Code, through the Biopython package. To obtain a high-quality core proteome dataset, coding sequences were excluded if they did not contain a valid open reading frame, had an incomplete start or stop codon, or represented a truncated protein sequence. On this basis, nine of the 1169 core genes were excluded, yielding 1160 valid translated core protein sequences for downstream analysis. These protein sequences were then put together in a non-redundant FASTA file, which was used as an input file for the subsequent subtractive proteomics pipeline comprising homology screening, essentiality analysis, physicochemical characterization, subcellular localization prediction and immunoinformatics-based identification of vaccine candidates [57,59,60].

### 4.3. Identification of Non-Homologous Proteins and Exclusion of Host Cross-Reactivity

The core proteome was screened against the human reference proteins to detect significant similarity to human proteins, to reduce the risk of potential autoimmune responses and host cross-reactivity. Protein sequence homology analysis was carried out with the BLASTp tool using the UniProt human reference proteome (Proteome ID: UP000005640) [61,62]. The E-value used for sequence alignment was 1 × 10^−5^ and the proteins that had a sequence identity ≥ 30% with human proteins were excluded from further analysis. Proteins with <30% sequence identity and no significant similarity to the human proteome were kept as non-homologous proteins to be used in subtractive proteomics analyses. This protein set was then filtered to exclude non-homologous proteins and was later compared to the Database of Essential Genes (DEG) for essentiality analysis.

### 4.4. Essential Protein Identification

The non-homologous proteins were tested to select proteins that are related to the survival and physiological functions of *S. suis*. Essentiality analysis was carried out by BLAST searching against the bacterial compartment of the Database of Essential Genes (DEG), which contains validated essential genes from various bacteria [63]. Protein sequence alignments were done with an E-value < 1 × 10^−5^ [63]. The proteins with significant similarity to the proteins found in the DEG database were retained for further analysis, while the proteins that did not match significantly were discarded. The analysis of essential non-homologous proteins was then followed by gut microbiome homology analysis to identify candidate vaccine proteins against pathogens.

### 4.5. Subcellular Localization and Transmembrane Helix Prediction

The computational tools PSORTb version 3.0.3 and CELLO v.2.5, which were developed to predict the localization of bacterial proteins, were used to analyze the proteins that passed the subtractive proteomics filtering criteria for subcellular localization analysis [64,65]. The protein sequences were divided into cytoplasmic, cytoplasmic membrane, cell wall, extracellular and unknown localization categories using an integrated prediction algorithm in PSORTb. Proteins predicted to be localized in the extracellular region or associated with the cell wall were selected for further analysis because of their accessibility to the host immune system.

The selected proteins were then subjected to TMHMM v.2.0 analysis to predict the number and position of the transmembrane helices [66]. Proteins that had a maximum of one transmembrane helix were kept for further analysis so that they could be expressed recombinantly and would be more suitable as vaccine candidates. The antigenic potential of the proteins selected was then evaluated with VaxiJen version 2.0, with the bacterial model. An antigenicity threshold of 0.5 was used and proteins showing an antigenicity score above the threshold value were considered potential vaccine candidates and used for epitope prediction [67].

### 4.6. Cytotoxic T Lymphocyte (CTL) Epitope Prediction and Screening

The selected antigenic protein sequences were submitted to the MHC class I binding prediction tool in the Immune Epitope Database (IEDB) Analysis Resource to identify Cytotoxic T lymphocyte (CTL) epitopes [68]. The epitope prediction was carried out by using the IEDB-recommended prediction method, which combines the outputs from several prediction algorithms to increase the accuracy of the peptide–MHC binding affinity prediction. Human MHC class I allele binding affinity and percentile ranking were used to select candidate epitopes. Candidate CTL epitopes were selected using a consensus percentile score below 2.0, followed by evaluation of their antigenicity, allergenicity, toxicity, and immunogenicity.

The predicted CTL epitopes were then tested for antigenicity with VaxiJen version 2.0, toxicity with ToxinPred and allergenicity with AllerTOP version 2.0. The multi-epitope vaccine construct was limited to the epitopes predicted to be antigenic, non-allergenic and non-toxic [69,70,71].

### 4.7. Helper T Lymphocyte (HTL) Epitope Prediction and Evaluation

The MHC class II binding prediction tool at the IEDB Analysis Resource was used to predict helper T lymphocyte (HTL) epitopes [68,72]. Peptides of 15 amino acids were tested for binding to the reference set of class II human HLA alleles using the IEDB-recommended prediction algorithm. Sequences with high binding affinity and low percentile rank were prioritized. Candidate HTL epitopes were selected based on a percentile rank below 2.0, followed by assessment of antigenicity, allergenicity, toxicity, and immunogenicity.

Selected HTL epitopes were subsequently checked for antigenicity with VaxiJen version 2.0, allergenicity with AllerTOP version 2.0 and toxicity with ToxinPred. Epitope(s) that met all of the screening standards were retained for vaccine construction [69,70,71].

### 4.8. Linear B-Cell Epitope Prediction and Screening

The selected vaccine candidate proteins were subjected to prediction of linear B-cell epitopes using the ABCpred server, which is an artificial neural network (ANN)-based prediction tool for the identification of linear B-cell epitopes from protein sequences [73]. The default window length of 16 amino acids and a threshold score of 0.51 were used in the prediction. Predicted epitopes with a score ≥ the selected value were considered potential B-cell epitopes and kept for further assessment.

The predicted B-cell epitopes were then validated for their antigenic properties using VaxiJen version 2.0 with a threshold of 0.5. AllerTOP version 2.0 was used to assess the allergenic properties, whereas ToxinPred was used to assess the toxic properties [69,70,71]. Only predicted epitopes that were antigenic, non-allergenic and non-toxic were chosen to be included in the final multi-epitope vaccine construct.

### 4.9. Population Coverage Analysis

The possible population coverage of selected CTL and HTL epitopes was predicted with the Population Coverage tool in the Immune Epitope Database (IEDB) [26]. Those HLA alleles that were shortlisted for the MHC class I and MHC class II (HLA class) epitopes were entered to estimate the percentage of the world’s population that would have an immune response to the proposed vaccine. The population coverage was determined from the distribution and frequencies of HLA alleles from various regions and ethnic groups listed in the IEDB database. To assess the potential applicability of the designed multi-epitope vaccine, global and regional population coverage analyses were carried out.

### 4.10. Multi-Epitope Vaccine Construct Design

The final designed multi-epitope vaccine (MEV) is a sequential concatenation of the selected CTL, HTL and linear B-cell epitopes. The immunogenicity of the construct was improved by using the 50S ribosomal protein L7/L12 as an adjuvant at the N-terminal end of the vaccine sequence. The adjuvant was conjugated to the first CTL epitope with an EAAAK linker in order to ensure structural rigidity and to prevent any undesirable interactions between the adjuvant and the epitopes [52,74].

The individual CTL epitopes were linked together with AAY linkers to aid in proteasomal processing and MHC class I presentation. The linear B-cell epitopes were separated by KK linkers to maintain their antigenicity and flexibility, while the HTL epitopes were linked together by GPGPG linkers for efficient processing and presentation via MHC class II molecules [75,76]. The final vaccine construct comprised all the components (epitope sequences, linkers and adjuvant) in a single chimeric protein sequence.

### 4.11. Immunological and Physicochemical Characterization of the Vaccine Construct

A server called ExPASy was used to examine the physicochemical properties of the designed vaccine construct with the help of the ProtParam tool [77]. Biochemical properties of the vaccine construct were estimated by calculating parameters such as molecular weight, theoretical isoelectric point (pI), instability index, aliphatic index, grand average of hydropathicity (GRAVY), estimated half-life, and amino acid composition.

The antigenicity of the complete sequence of the vaccine was assessed with VaxiJen version 2.0 and the bacterial prediction model. AllerTOP version 2.0 was used for assessing allergenicity and ToxinPred was used for assessing toxicity. Immunogenic potential was also evaluated by running the vaccine construct through the IEDB immunogenicity prediction tool [67,68,70,71].

### 4.12. Secondary and Three-Dimensional Structure Prediction, Refinement, and Validation

The secondary structural features of the designed multi-epitope vaccine were predicted with the help of the Self-Optimized Prediction Method with Alignment (SOPMA) server [78]. The proportions of alpha-helices, beta-strands, beta-turns and random coils in the vaccine sequence were also predicted. Four conformational states, a window width of 17 and a similarity threshold of 8 were used, along with default prediction parameters. The three-dimensional structure modeling was performed after the prediction of secondary structural composition.

The 3D structure of the multi-epitope vaccine construct was predicted with the aid of AlphaFold2, a deep-learning-based model that can produce highly confident 3D models of proteins directly from their amino acid sequences [79]. The predicted structural model was then processed using the GalaxyRefine server for the improvement of backbone geometry, optimization of side-chain conformations and general improvement of the stereochemical quality of the protein model [80,81].

Several structural validation tools were used to assess the quality of the refined three-dimensional structure. The stereochemical quality of the predicted model was evaluated by analyzing the distribution of amino acid residues in energetically favorable conformational regions using Ramachandran plot analysis performed with RAMPAGE [82]. Structural reliability was assessed additionally by using the ERRAT server to analyze non-bonded atomic interactions and identify potential structural inconsistencies [83]. The validated structural model was then used for conformational epitope prediction and molecular docking analyses.

### 4.13. Predicting Conformational B-Cell Epitopes

Conformational B-cell epitopes of the multi-epitope vaccine construct were discovered by analyzing its refined three-dimensional structure with the ElliPro server of the Immune Epitope Database (IEDB) [84]. The discontinuous structure of the epitope is predicted by ElliPro, considering the position of the amino acids and the values of the protrusion index calculated from the tertiary structure.

### 4.14. Molecular Docking with Immune Receptors

A molecular docking approach was used to investigate the binding interactions of the designed multi-epitope vaccine construct with the immune receptor molecules. The 3D structures of the vaccine construct and the selected immune receptors were created and then submitted to the ClusPro 2.0 server for the docking of the two proteins together [85]. To achieve this, ClusPro was used for rigid-body docking, clustering of the lowest-energy docking poses and energy minimization to generate biologically relevant docking models. Energy-weighted score and cluster size were used to rank the docked complexes, and the top-ranked complexes were chosen for further analysis [25].

The intermolecular interactions between the vaccine construct and the immune receptors, such as hydrogen bonds, salt bridges, hydrophobic interactions and interface residues, were evaluated with the PDBsum server [86]. In addition, the thermodynamic properties of the docked complexes were investigated on the PRODIGY server [87]. To further evaluate the stability and strength of the predicted interactions, the binding affinity (ΔG), dissociation constant (Kd) and other protein–protein interface-related thermodynamic parameters were estimated based on the structural features of the protein–protein complexes.

### 4.15. Normal Mode Analysis (NMA)

Furthermore, the intrinsic structural flexibility and dynamic behavior of the docked vaccine receptor complex were assessed with the use of the iMODS web server, which is based on Normal Mode Analysis (NMA) in internal coordinates and allows the prediction of large-scale collective motions of biomolecular complexes with reduced computational cost [88]. The refined docked complex from molecular docking was then sent to the iMODS server for dynamic analysis.

Normal mode analysis was used to study the collective motion, conformational flexibility and the structural stability of the complex. The default parameters of the iMODS server were used to calculate several dynamic properties such as B-factor, deformability, eigenvalue, variance, covariance matrix, and elastic network model. The residue flexibility was estimated using the B-factor profile, and the potential hinge regions involved in conformational changes were identified with the help of the deformability plot. The smaller the eigenvalue, the greater the structural mobility, and the less energy was required to deform the complex. The individual normal modes were measured and their contribution to the overall molecular motion, using variance analysis, was determined. Correlated, anti-correlated and uncorrelated movement of the residues was assessed throughout the complex using the covariance matrix, while the elastic network model revealed the mechanical coupling and interaction strength of residue pairs, offering insights into the structural rigidity and stability of the vaccine–receptor complex [89].

### 4.16. Molecular Dynamics (MD) Simulation

The structural stability and dynamic behavior of the vaccine–receptor complex were assessed by molecular dynamics (MD) simulation using the Schrödinger Suite (Version 2025-4) (Schrödinger, LLC, New York, NY, USA) [90]. The docked complex was prepared for simulation by the Protein Preparation Wizard, in which various hydrogen bonding networks were optimized, bond orders were assigned, missing H atoms were added, and restrained energy minimization was performed.

The optimized system was parameterized with the OPLS-2005 force field [91]. To simulate the complex, the distance between the solute and the boundaries of the box was kept to at least 10 Å, and the complex was solvated in an orthorhombic simulation box in which explicit TIP3P water molecules were present. To obtain the physiological ionic environment, the required numbers of Na^+^ and Cl^−^ ions were added to achieve electrical neutrality and physiological conditions. The default multi-step equilibration protocol offered by Desmond was followed on the energy-minimized structure to eliminate any unfavorable steric interactions and ensure the stability of the system prior to the production run [92].

The production run was performed for 200 ns under the NPT ensemble with a constant temperature of 310 K and a constant pressure of 1 atm. A series of trajectory frames were captured at regular intervals during the simulation for later analysis. To assess the structural stability and conformational flexibility of the complex, the root mean square deviation (RMSD) and root mean square fluctuation (RMSF) of the complex were calculated. Additionally, the binding free energy of the vaccine-receptor complex was calculated by the Molecular Mechanics Generalized Born Surface Area (MM-GBSA) method using representative snapshots from the simulations [93].

### 4.17. Immune Simulation

The immunogenic potential of the designed multi-epitope vaccine was assessed in the C-ImmSim server version 10.1 [94]. Immune system parameters of the default mammalian system were used in the simulation and the selected human leukocyte antigen (HLA) alleles represented MHC class I and MHC class II molecules. The simulation was configured to model vaccine administration and forecast the subsequent immune response for multiple simulation steps. The server was used to estimate antigen clearance, antibody production, cytokine secretion, activation of B lymphocytes, helper T lymphocytes, cytotoxic T lymphocytes, macrophages, dendritic cells and generation of memory immune cells [95].

### 4.18. In Silico Cloning

The *in silico* cloning of a multi-epitope vaccine construct was performed to test its suitability for recombinant expression in *E. coli*. For optimal translation in *E. coli* (strain K12), the Java Codon Adaptation Tool (JCat) was used for codon optimization. Codon adaptation index (CAI) and GC content were evaluated for compatibility with the host expression system, with conditions of a codon adaptation index value close to 1.0 and GC content between 30 and 70%, indicating good expression potential [96].

The molecular biology platform Benchling was used for *in silico* cloning [97]. The codon-optimized sequence of the vaccine gene was designed with EcoRI and HindIII restriction enzyme sites at the 5′ and 3′ ends, respectively, to ensure that the gene was inserted in the proper orientation in the chosen expression vector. The insert was placed into the vector at the multiple cloning site (MCS) with the proper orientation and reading frame for downstream protein expression using the Benchling cloning workflow. The recombinant plasmid construct was then checked *in silico* to ensure that the vaccine sequence was correctly inserted and that key elements that are necessary for efficient transcription and translation were retained.

### 4.19. Data Analysis and Statistical Considerations

All computational analyses were performed using a combination of established bioinformatics tools, web servers, and custom Python scripts. Unless otherwise specified, the default parameters recommended by each software package or web server were applied. Selection thresholds for protein and epitope screening were based on the recommended criteria of the respective tools and supported by previously published reverse vaccinology studies. The results were summarized and visualized using appropriate graphical software, and no inferential statistical analyses were performed because the study was entirely computational.

### 4.20. Use of Generative Artificial Intelligence

During the preparation of this manuscript, the authors used ChatGPT (OpenAI) solely for language editing, writing refinement, and improving the clarity and readability of the text. Generative AI was not used to generate or fabricate research data, conduct data collection or analysis, generate scientific results, create or manipulate figures, determine the study design, or independently interpret the study findings. All AI-assisted output was carefully reviewed, verified, and edited by the authors, who take full responsibility for the originality, accuracy, validity, integrity, and final content of this publication.

## 5. Conclusions

In this study, we developed a pangenome-guided reverse vaccinology and immunoinformatics framework to identify and prioritize conserved vaccine targets against diverse *S. suis* strains. Comparative analysis of 24 complete genomes identified a conserved core genome that provided a rational basis for systematic antigen discovery, while sequential subtractive proteomics and reverse vaccinology prioritized three candidate proteins—sucrose phosphorylase, peptidoglycan hydrolase PcsB, and an RND transporter-associated adaptor protein, annotated in the source database as an RND efflux transporter periplasmic adaptor subunit—as putative vaccine targets. Integrating experimentally relevant selection criteria with CTL, HTL, and B-cell epitope prediction enabled the rational construction of a 397-amino-acid multi-epitope vaccine incorporating the *S. suis* 50S ribosomal protein L7/L12 adjuvant and optimized peptide linkers. The resulting construct exhibited favorable predicted antigenicity, physicochemical characteristics, structural quality, and a predicted HLA population coverage of 90.77% across the populations evaluated. Molecular docking and 200 ns molecular dynamics simulations further supported a computationally predicted interaction between the vaccine construct and TLR1/TLR2, while immune simulations predicted coordinated humoral and cellular responses, including antibody production, cytokine secretion, and immunological memory. In addition, codon optimization and *in silico* cloning indicated favorable prospects for recombinant expression in *Escherichia coli*. Importantly, the proposed vaccine represents a computationally prioritized candidate rather than an experimentally validated vaccine. Its principal strength lies in the integration of pangenomic conservation with multi-layered antigen, structural, immunological, and expression analyses, thereby providing a systematic strategy for addressing the antigenic diversity and diverse strains of *S. suis*. The selected epitopes were derived from proteins belonging to the conserved core proteome identified across the analyzed *S. suis* genomes. This provides evidence that the corresponding vaccine-target proteins are broadly represented within the analyzed dataset. Nevertheless, core-protein conservation does not necessarily imply complete conservation of individual epitope sequences, and residue-level conservation of the selected epitopes was not independently assessed in the present study. The inclusion of WP_256768021.1 provides an additional computationally prioritized antigen candidate; however, its precise localization, functional role, and suitability as a vaccine target require experimental characterization. Nevertheless, the predicted antigenicity, receptor engagement, structural stability, and immune responses require rigorous experimental confirmation. Future studies should therefore evaluate recombinant expression and purification, antigenicity and receptor interaction, cellular immune activation, and, ultimately, safety, immunogenicity, cross-serotype protection, and protective efficacy in appropriate animal models. Overall, the present findings provide a rational and experimentally testable foundation for advancing this multi-epitope vaccine candidate toward laboratory validation and, potentially, the development of a broadly protective strategy against diverse *S. suis*.

## Figures and Tables

**Table 1:** Proteins identified by a subtractive proteomics approach in *S. suis* as its final vaccine candidate proteins. The shortlisted proteins with their antigenicity, allergenicity and toxicity profiles are summarized in the table.

Accession No.	Protein	Antigenicity	Allergenicity	Toxicity
WP_174851906.1	Sucrose phosphorylase	0.5927	Non-allergen	Non-toxin
WP_256768021.1	RND transporter-associated adaptor protein (annotated in the source database as efflux RND transporter periplasmic adaptor subunit)	0.6414	Non-allergen	Non-toxin
WP_136628818.1	Peptidoglycan hydrolase PcsB	0.6262	Non-allergen	Non-toxin

**Table 2:** The predicted cytotoxic T lymphocyte (CTL) epitopes of the selected vaccine candidate proteins of *S. suis*. The table shows the short-listed epitopes, their source proteins, the HLA class I alleles, their positions in the protein, their antigenicity and immunogenicity.

Epitope	Protein	Allele	Position	Antigenicity	Immunogenicity
YYLMFDFMINHI	Sucrose phosphorylase	HLA-A*02:01	77–88	2.141	0.03702
YVYDFALPMLVL	Sucrose phosphorylase	HLA-A*02:01 HLA-C*07:02	251–262	1.7267	0.00108
ATGTSGGSGAKY	RND transporter-associated adaptor protein	HLA-A*30:02 HLA-A*01:01	274–285	2.9274	−0.26722
TGTSGGSGAKYP	RND transporter-associated adaptor protein	HLA-A*30:02	275–286	2.6191	−0.3633
GTSGGSGAKYPF	RND transporter-associated adaptor protein	HLA-A*30:02 HLA-B*35:01	276–287	2.5737	−0.2892
SGSSAAANNARY	Peptidoglycan hydrolase PcsB	HLA-A*30:02 HLA-A*01:01	295–306	1.2942	−0.055
GSSAAANNARYD	Peptidoglycan hydrolase PcsB	HLA-A*30:02 HLA-B*07:02	296–307	1.2859	0.12762
FNPTTTSEGAVY	Peptidoglycan hydrolase PcsB	HLA-A*01:01 HLA-A*24:02	401–412	1.1096	0.1365

An asterisk (*) is part of the standard HLA allele nomenclature and separates the HLA gene name from its allele designation.

**Table 3:** Selected vaccine candidate proteins of *S. suis* were used to predict helper T lymphocyte (HTL) epitopes. Selected HTL epitopes with their corresponding HLA class II alleles, peptide positions, antigenicity and immunogenicity scores are shown in the table.

Epitope	Protein	Allele	Position	Antigenicity	Immunogenicity
EQDYYVYDFALPMLV	Sucrose phosphorylase	HLA-DRB1*03:09 HLA-DRB3*01:01 HLA-DRB1*03:05 HLA-DRB1*04:01 HLA-DRB1*04:26 HLA-DRB1*03:08	247–261	1.2834	0.03426
FVWTIVEGKAKKVEV	RND transporter-associated adaptor protein	HLA-DRB5*01:05 HLA-DRB1*11:01	230–244	1.3322	−0.0803
DTAVRGRDKIGRQIY	RND transporter-associated adaptor protein	HLA-DRB1*08:04 HLA-DRB1*13:01 HLA-DRB1*01:01	115–129	1.3196	0.19096
VQSSTSIQVVESNYM	Peptidoglycan hydrolase PcsB	HLA-DRB1*07:03 HLA-DRB1*04:10	374–388	0.8472	−0.31212
VTAVQSSTSIQVVES	Peptidoglycan hydrolase PcsB	HLA-DRB1*07:01 HLA-DRB1*07:03	371–385	0.6784	−0.35506

An asterisk (*) is part of the standard HLA allele nomenclature and separates the HLA gene name from its allele designation.

**Table 4:** Predicted linear B-cell epitopes of the selected vaccine candidate proteins of *S. suis*. The shortlisted epitopes, source proteins, ABCpred scores, peptide positions, antigenicity and immunogenicity values are summarized in the table.

Epitope	Protein	Score	Start Position	Antigenicity	Immunogenicity
FSVKMLDETSMEIFWN	Sucrose phosphorylase	0.79	464	0.7402	−0.1096
KITYISNYPSTEQTAS	RND transporter-associated adaptor protein	0.63	311	0.5027	−0.12254
MREIVSANNRMLEQQK	Peptidoglycan hydrolase PcsB	0.86	178	0.5496	−0.13157

## Data Availability

The original contributions presented in this study are included in the article/Supplementary Material. Further inquiries can be directed to the corresponding authors.

## Supplementary Materials

The following supporting information can be downloaded at: https://www.mdpi.com/article/10.3390/ph19091448/s1, Table S1: NCBI assembly and annotation metadata for the complete *Streptococcus suis* genomes used in the pangenome analysis. The table lists the strain designation, RefSeq assembly accession number, annotation source, assembly level, and assembly release date.

## Abbreviations

The following abbreviations are used in this manuscript:

AMR	Antimicrobial Resistance
BLASTp	Basic Local Alignment Search Tool for Proteins
CAI	Codon Adaptation Index
CTL	Cytotoxic T Lymphocyte
DEG	Database of Essential Genes
HLA	Human Leukocyte Antigen
HTL	Helper T Lymphocyte
IEDB	Immune Epitope Database
IFN-γ	Interferon-Gamma
IgG	Immunoglobulin G
IL-2	Interleukin-2
MD	Molecular Dynamics
MDR	Multidrug Resistance
MEV	Multi-Epitope Vaccine
MHC	Major Histocompatibility Complex
NMA	Normal Mode Analysis
RND	Resistance-Nodulation-Division
RMSD	Root Mean Square Deviation
RMSF	Root Mean Square Fluctuation
TLR1/TLR2	Toll-Like Receptor 1/2
